# Comparative Mitogenomics Reveals Intron Dynamics and Mitochondrial Gene Expression Shifts in Domesticated and Wild *Pleurotus ostreatus*

**DOI:** 10.3390/jof12010075

**Published:** 2026-01-20

**Authors:** Gumer Pérez, Idoia Jiménez, Edurne Garde, Lucía Ramírez, Antonio G. Pisabarro

**Affiliations:** Genetics, Genomics and Microbiology Research Group, Institute for Multidisciplinary Research in Applied Biology (IMAB), Public University of Navarre (UPNA), 31006 Pamplona, Spain; idoia.jimenez@unavarra.es (I.J.); edurne.garde@unavarra.es (E.G.); lramirez@unavarra.es (L.R.)

**Keywords:** mitochondrial genomes, basidiomycetes, homing endonuclease, genome reduction

## Abstract

Mitochondrial genomes play a central role in fungal physiology and adaptation, yet their evolutionary dynamics during domestication remain poorly understood. Here, we performed a comparative mitogenomic and gene-expression analysis of three *Pleurotus ostreatus* dikaryotic strains differing in origin and degree of adaptation to laboratory conditions: the long-term commercial strain dkN001, the laboratory-maintained wild isolate dkF515, and the recently collected wild strain dkN009. High-throughput Illumina sequencing enabled complete assembly of circular mitochondrial genomes, revealing substantial size variation among strains, where the dkN001 strain exhibited the second smallest mitogenome reported for the genus *Pleurotus*. Comparative analyses showed >99% sequence identity between wild isolates and ~95% identity relative to the commercial strain. Variations in genome size among strains were associated with intron dynamics in the *cox1* and *rnl* genes, as well as intron loss predominantly in the commercial strain dkN001, consistent with mitochondrial genome streamlining during domestication. Expression profiling of mitochondrial protein-coding genes (PCGs) under multiple culture conditions revealed conserved transcriptional responses in dkN001 and dkF515 that contrasted sharply with those of dkN009. The differences observed, which affected components of the electron transport chain, suggested shifts in energy metabolism associated with long-term laboratory maintenance. Therefore, our results demonstrate that domestication in *P. ostreatus* involves both structural remodelling of the mitogenome and changes in regulation of mitochondrial PCGs, highlighting the importance of mitonuclear interactions in fungal adaptation to controlled environments.

## 1. Introduction

Mitochondria are mainly responsible for cellular respiration and contain their own genomes. Mitochondrial genomes (mitogenomes) are involved in a wide range of physiological and biochemical processes, including cellular growth and development, stress response, and energy metabolism, among others [1,2]. Throughout evolution, most mitochondrial genes are transferred to the nucleus; consequently, the majority of mitochondrial proteins are nuclear-encoded, translated by cytosolic ribosomes, and subsequently imported into the mitochondria [3].

Crosstalk between nuclear and mitochondrial genomes, referred to as mitonuclear interaction, is key for the proper regulation of oxidative energy production through the Electron Transport Chain (ETC) located in the inner membrane of this organelle. The correct function of the ETC depends on the coordinated expression of both nuclear- and mitochondrially encoded proteins and plays a role in regulating cellular metabolism, as well as the production of reactive oxygen species generated by electron flow through the respiratory chain [4,5,6,7].

In fungi, most core components of oxidative phosphorylation are encoded in the mitogenomes. This includes the seven protein-coding genes (PCGs) in the mitogenome of *Saccharomyces cerevisiae*, the thirteen PCGs in *Cryptococcus neoformans*, and the fourteen PCGs found in certain species of the genera *Aspergillus*, *Penicillium*, and *Pleurotus*. These genes exhibit a highly conserved synteny among most fungal species [8,9]. The size of fungal mitogenomes varies considerably [10,11], largely due to differences in intron number and size, the presence of repetitive elements, and the acquisition of new genes through horizontal gene transfer [12]. Mitochondrial introns in fungi are classified into two groups—group I and group II—based on their RNA secondary structures involved in self-splicing mechanisms [13]. These introns contribute to variations in mitogenome organisation and size [12,14] and have been identified in *cob*, *nad*, *cox*, *rnl* and *rns* genes of fungal mitogenomes [15]. Most fungal mitochondrial introns belonging to group I harbour Open Reading Frames (ORFs) encoding Homing Endonucleases (HEs) with GIY-YIG (GIY) and LAGLIDADG (LD) motifs [15,16]. Organelle introns are generally considered mobile genetic elements driven by intronic HEs. They have been described as “trans-genomic” invasive introns that contribute to structural rearrangements and size variation in mitogenomes [11].

*Pleurotus ostreatus*, commonly known as the oyster mushroom, is an excellent model for studying mitochondrial domestication because it combines a well-characterised genome, the availability of both wild and commercially cultivated strains, and a long history of laboratory maintenance, allowing the disentangling of evolutionary changes driven by natural variation, artificial selection, and controlled growth conditions. It is an edible filamentous fungus and one of the most widely cultivated mushrooms worldwide, alongside other commercially important species such as *Agaricus bisporus* and *Lentinus edodes* [17,18,19]. It is highly valued for its nutritional composition and diverse biotechnological applications [20,21]. The *P. ostreatus* dkN001 strain, which exhibits a tetrapolar mating system with four distinct mating types generated through meiosis, is employed in our laboratory as a model organism for studying diverse biological processes [22,23,24], where the most recent research by our group has shown that incompatible mitonuclear combinations exhibit reduced growth rates and enhanced expression of genes associated with the ETC and antioxidant defences [25].

Mitochondrial adaptations rely on conserved mechanisms, such as the regulation of PCG expression, that modulate mitochondrial respiration to preserve cellular and organismal homeostasis under fluctuating environmental conditions [26]. Previous studies have demonstrated that various organisms can adapt to local [27,28] or to laboratory environmental conditions [29] through natural selection based on genetic variation, or via phenotypic plasticity.

Accordingly, the main objectives of this study were (1) to analyse the organisation of the mitogenomes in three *P. ostreatus* strains (dkN001, dkN009, and dkF515), considering their origin (commercial or wild) and maintenance conditions; (2) to provide insights into the evolution and dynamic changes of introns within the mitogenomes of the species belonging to the *Pleurotus* genus; and (3) to assess the capacity of mitochondrial PCGs to alter their expression levels under different culture conditions analysed, both within and among strains.

## 2. Materials and Methods

### 2.1. Fungal Strains and Growth Conditions

Three dikaryotic strains of *P. ostreatus* have been used in this study. The commercially (domesticated) dikaryotic strain dkN001 (Spanish Type Culture Collection accession CECT20600) has been maintained in our laboratory under Standard culture Conditions (SC) for more than 25 years, undergoing approximately 15–20 subcultures per year. The dikaryotic wild strain dkF515 was isolated in Colombia in 2009 and has since been maintained under the same laboratory conditions as strain dkN001. Consequently, both dkN001 and dkF515 dikaryotic strains are considered to be adapted to laboratory culture conditions. The dikaryotic wild strain dkN009 was isolated from Alcalá de Henares (Madrid, Spain) in 2019 and has been maintained with one or two subcultures per year. In this study, dkN009 is considered a wild-type strain not adapted to laboratory conditions. All strains were grown in Standard Conditions on Petri dishes with either Malt Extract Solid Medium (MESM: malt extract, 20 g/L; and bacteriological agar, 15 g/L) or in Minimal Solid Medium (MSM: 0.1 g/L Na_2_B_4_O_7_·H_2_O, 0.07 g/L ZnSO_4_·7H_2_O, 0.01 g/L CuSO_4_·5H_2_O, 0.01 g/L MnSO_4_·4H_2_O, 0.01 g/L FeSO_4_·4H_2_O, 0.01 g/L (NH_4_)6Mo_7_O_2_·4H_2_O and 15 g/L bacteriological agar) supplemented with saccharose (4 g/L) or glycerol (4 mL/L). All cultures were performed at 24 °C, unless otherwise indicated. Dikaryotic strains were grown on Petri dishes until they reached the edge under dark conditions. To measure growth rate, five biological replicates were used, and three of them were employed for qPCR experiments.

### 2.2. Nucleic Acid Extractions, Sequencing and Assembly of Mitochondrial Genome

Mycelia obtained in all conditions (MESM at 15 °C; MESM at 24 °C; MEMS at 32 °C; MSM supplemented with saccharose, and MSM supplemented with glycerol) were harvested from the surface of Petri dishes with a sterile scalpel, ground in a sterile mortar using liquid nitrogen, and stored at −80 °C. Nuclear and mitochondrial DNA was extracted using the protocol described by [22]. To obtain total RNA, the fungal RNA E.Z.N.A. kit (Omega Bio-Tek, Norcross, GA, USA) was used. The sequencing was performed with an Illumina NovaSeq6000 system (Illumina, San Diego, CA, USA) using 150 bp paired-end reads. The MITObim programme (included in the Galaxy web platform), an available reference-based assembler, was used to assemble mitochondrial genomes of this work. The wrapper programme mitoBIM uses MIRA to reconstruct mitochondrial genomes [30]. Using MIRA in mapper mode, it first baits perceived mitochondrial reads from the whole DNA sequence with known mitochondrial genes and/or genomic sequences and then uses the MIRA in assembler mode to put those readings together. *P. ostreatus* strain mkPC15 mitogenome, retrieved from Join Genome Institute, was used as the reference and seed to bait the mitogenome sequences from other strains (Retrieved from https://mycocosm.jgi.doe.gov/PleosPC15_2/PleosPC15_2.home.html; accessed on 12 January 2024).

### 2.3. Gene Annotation and Bioinformatics Analysis

Mitogenome sequences for three *P. ostreatus* strains were checked for homology in the NCBI BLASTn algorithm version 2.16.0 (released 25 June 2024). Mitogenome sequences of three strains were annotated using MFannot programme based on the genetic code 4 (http://megasun.bch.umontreal.ca/cgi-bin/mfannot/mfannotInterface.pl accessed on 22 December 2025) [31]. Putative secondary structures of transfer RNAs (tRNAs) were determined using MITOS web server integrated into the Galaxy platform [32] and the RNAfold bioinformatic tool [33]. Open Reading Frames (ORFs) were analysed by homology using BLASTx tool in the NCBI version 2.16.0 (released 25 June 2024). GenBank database (http://www.ncbi.nlm.nih.gov accessed on 22 December 2025). The data obtained data with the MFannot programme were converted into .GBF files to construct the mtDNA maps, which were visualised by OGDraw (http://chlorobox.mpimp-golm.mpg.de/OGDraw.html, accessed on 20 December 2025) [34]. The mitogenome sequences were alignments to identify syntenic blocks between dkN001, dkN009 and dkF515 strains using MAUVE 2.4.0 [35]. Sequence logo representation of amino acid conservation in the LAGLIDADG motifs found in intronic regions of *cox1* gene was generated using WebLogo [36]. To investigate the presence of intra-genomic duplications of large fragments or interspersed repeats within the strains’ mtDNAs, we performed BLASTn analysis version 2.17.0 (released 22 July 2025) (e-value 10–10) by comparing the mitogenomes against themselves. We used Tandem Repeat Finder (v4.09; https://tandem.bu.edu/trf/trf.html, accessed on 20 December 2025) with default parameters to identify tandem repeats longer than 10 bp [37].

### 2.4. Analysis of Gene Expression

The relative expression level of genes used in this study was analysed by quantitative reverse transcription PCR (RT-qPCR). Total RNA (800 ng per sample) was reverse transcribed into cDNA using an iScript cDNA synthesis kit (Bio-Rad Laboratories Inc., Hercules, CA, USA). Reverse transcription (RT) was carried out in a thermal cycler (MJ Research Inc., Waltham, MA, USA) following the manufacturer’s instructions. RT-qPCRs were performed in a CFX96 real-time system (Bio-Rad Laboratories Inc.) using SYBR green dye to detect product amplification. Each reaction mixture contained 10 μL iQ SYBR green supermix (Bio-Rad Laboratories Inc.), 2 μL of each primer (3 μM, forward and reverse), 1 μL of a 1:20 dilution of the RT product, and 5 μL of sterile water. The amplification programme consisted of 5 min at 95 °C and 40 cycles of 15 s at 95 °C, 30 s at 63 °C, and 15 s at 72 °C. Three biological replicates were used for each sample. Relative gene expression was determined by the 2^−ΔΔCt^ method using the GenEx5 software for processing and analysis of qPCR data. The qPCR data were normalised using three reference genes (*sar1*, *sar4* and *sar5*) which encode small GTPases [38]. Data were analysed using IBM SPSS Statistics version 27.0 (IBM Corp., released 2020; Armonk, NY, USA). One-way ANOVA was applied to determine significant differences, followed by Scheffé’s post hoc test to compare mean values for intra-strain and inter-strain differences under different culture conditions. All Primer pairs used in this work are shown in Table S1.

### 2.5. Data Availability

The mtDNA of dkN001, dkN009 and dkF515 strains analysed in this study has been deposited in GenBank (https://ncbi.nlm.nih.gov accessed on 22 December 2025) under the accession numbers PX724300, PX724302 and PX724301, respectively.

## 3. Results

The dkN001 strain used in this study is a commercial cultivar that has been maintained under laboratory conditions through continuous subculture since 1998. Such prolonged maintenance has resulted in strain degeneration or hybrid breakdown, manifested as a decline in agronomic traits such as growth rate and yield [24,25]. In contrast, dkF515 and dkN009 are wild isolates collected in 2009 and 2019, respectively, and preserved using different methods, as described in Section 2. For the purposes of this study, dkN001 and dkF515 are considered long-term laboratory-maintained strains of different origin (commercial vs. wild) and are compared with the most recent wild-type isolate, dkN009.

### 3.1. Mitochondrial Genome Overview of P. ostreatus dkN001, dkN009 and dkF515 Strains

**Figure 1:**
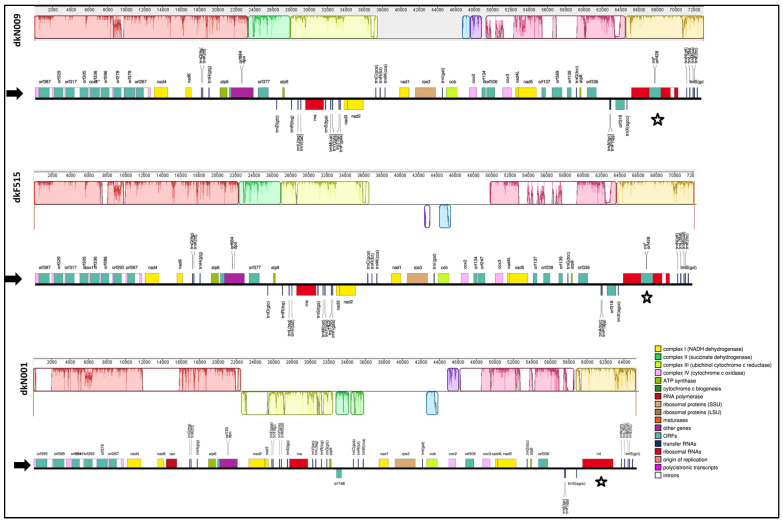
Colinearity analysis of the dkN009, dkF515, and dkN001 mitogenomes performed using Mauve v2.4.0, starting from the *cox1* gene. Colinear blocks are indicated with the same colour, and blocks below the centre line represent inversions. Corresponding linear physical maps of the mitogenomes (shown with arrows) were generated to illustrate the positions of protein-coding genes, which are represented by boxes of different colours. The *rnl* gene is indicated by a star and genes containing intron sequences are indicated by the symbol *. The figure was generated using OGDRAW version 1.3.1.

Complete circular mitochondrial genomes were assembled for three dikaryotic strains (dkN001, dkN009, and dkF515) of *P. ostreatus* using high-throughput Illumina sequencing data. The high sequencing depth, exceeding 20,000×, enabled the accurate reconstruction of full mitochondrial genome sequences for all strains (Table S2). Variation in mitogenome size was observed among the assemblies, with more than 7700 base pairs (bp) separating the largest (dkN009) and smallest (dkN001) genomes, indicating that the mitogenome of dkN001 was approximately 10.6% smaller than that of dkN009. The dkN001 mitogenome, with 65,303 bp, was the smallest and corresponds to a commercial cultivar maintained in laboratory conditions for nearly 30 years. The next in size was the mitogenome of the strain dkF515 (72,132 bp), which has been maintained under the same laboratory conditions as dkN001 for more than 15 years. Lastly, the wild-type strain dkN009 possessed the largest mitogenome with 73,085 bp (Figure 1). Comparative analysis of the mitochondrial genomes among the strains revealed a nucleotide sequence identity greater than 99% between the wild isolates dkN009 and dkF515, and approximately 95% between these isolates and the commercial strain dkN001. Sequence identity percentages were calculated using BLAST alignments between the mitogenomes; therefore, only the regions that could be aligned were considered in the calculation, meaning that the reported values reflect similarity in conserved regions rather than across the entire genome. Given the differences in genome size among strains, the overall genome-wide identity would be lower. Both the AT and GC skew in all strain mitogenomes were positive, where the AT content was 73.3% in strains dkF515 and dkN009 and 73.8% in dkN001.

Comparative genomic analyses between the mitogenomes obtained in this study and those publicly available in the NCBI database revealed that the mitochondrial genome of commercial strain dkN001 is the second smallest reported within the *Pleurotus* genus, following *P. citrinopileatus* (60,694 bp; accession number MG017444). Notably, the dkN001 mitogenome is 7939 bp shorter than that of the *P. ostreatus* P51 strain available in the NCBI database (accession number NC_009905).

Protein-Coding Gene (PCG) models and intronic regions within the mitochondrial genomes were predicted, identified, and classified using the MFannot tool. Consistent with most fungal species, all analysed *P. ostreatus* strains contained the full set of 15 conserved core PCGs commonly present in fungal mitogenomes. These included seven subunits of NADH dehydrogenase (*nad1*, *nad2*, *nad3*, *nad4L*, *nad4*, *nad5*, and *nad6*); three cytochrome c oxidase subunits (*cox1*, *cox2*, and *cox3*); *cytochrome b* (*cob*); *ribosomal protein S3* (*rps3*); and three ATP synthase subunits (*atp6*, *atp8*, and *atp9*). Furthermore, as reported in several fungal genera, the *nad4L* gene overlapped with *nad5* by a single nucleotide at their respective stop and start codons. In addition, 11, 17, and 18 open reading frames (ORFs) were predicted in the dkN001, dkF515, and dkN009 strains, respectively, using the MFannot tool (https://megasun.bch.umontreal.ca/apps/mfannot/ accessed on 22 December 2025) (Figure 1).

In the mitogenomes of the wild isolates dkF515 and dkN009, the 15 conserved PCGs were sequentially arranged as follows: *cox1*–*nad4*–*nad6*–*atp6*–*atp9*–*nad3*–*nad2*–*nad1*–*rps3*–*cob*–*cox2*–*cox3*–*nad4L*–*nad5*–*atp8*. By contrast, the mitogenome of commercial strain dkN001 exhibited an inversion affecting approximately 13.7% of its mitochondrial genome. This inversion involved two complex I genes (*nad2* and *nad3*), one complex V gene (*atp9*), the small subunit ribosomal RNA (*rns*), and eight tRNAs, indicating complex gene rearrangements likely associated with this commercial strain.

All mitogenomes contained 24 tRNA genes, with individual gene lengths ranging from 71 to 86 bp. In all strains, the transfer of arginine, leucine, and serine is facilitated by two distinct tRNA genes for each amino acid, each corresponding to different codons. Notably, in the dkN001 strain, the two copies of the *trnL* gene recognise the CUA and UUG codons, whereas in the remaining strains, recognition occurs for the CUA and UUA codons. Methionine transfer is mediated by two slightly divergent copies of the *trnM* (CAU) gene, both of which recognise the CAU codon in all strains. Finally, all mitogenomes harbour a *trnX* (AGCC), which may represent a pseudogene (Table S3). This pattern could provide important insights into codon usage preferences within the mitochondria of *P. ostreatus* strains. With respect to the predicted secondary structures, several tRNAs—such as those for cysteine, isoleucine, leucine, serine, threonine, tryptophan, and tyrosine—exhibited notably elongated variable loops, resulting in a starfish-like shape in contrast to the conventional cloverleaf structure (Figure S1). In the mitogenomes, two ribosomal RNA genes were also identified: the large subunit ribosomal RNA (*rnl*) and the small subunit ribosomal RNA (*rns*). Intronic regions were detected within the *rnl* gene in the wild isolates *dkF515* and *dkN009*, but were absent in the commercial strain dkN001, as indicated by stars in Figure 1.

Comparative analysis of the mitogenomes reveals that, despite the presence of an inverted region distinguishing the commercial strain from its wild counterparts, the *P. ostreatus* mitogenomes exhibit a highly conserved gene order. Furthermore, a high degree of sequence identity was observed, with 99.3% identity between wild isolates (dkF515 and dkN009) and 95.4% identity between these isolates and the commercial strain dkN001.

### 3.2. Repetitive Elements in P. ostreatus Mitogenomes

Mitogenomes are generally acknowledged to contain various types of repeated sequences, including intragenomic duplications, tandem repeats and transposon elements [39,40]. We analysed the mitogenomes of *P. ostreatus* strains to identify and characterise their repetitive sequences. A total of 88 intragenomic duplications were identified, with a marked bias between the commercial strain dkN001 (8 duplications), and the wild isolates dkN009 and dkF515, which harboured 37 and 43 duplications, respectively. The length of these sequences ranged from 35 to 554 bp, with pairwise nucleotide similarities between 72.73% and 100%. The largest repeats were identified in the coding regions of *orf317* and *orf319* in both wild isolates, whereas these ORFs were absent in the commercial strain dkN001 (Table S4).

We also identified 53, 52 and 49 tandem repeat sequences in the dkN001, dkN009 and dkF515 mitogenomes, respectively (Table S5), using Tandem Repeat Finder. The longest tandem repeat sequence, with a consensus size of 141 bp, spanned the terminal region of the ***rns*** gene and the intergenic region between *rns* and *trnV* (UAC) genes in the wild isolate dkF515. Tandem repeats accounted for 3.52% of the mitogenome in both wild isolates, whereas in the commercial strain dkN001, they comprised 4.63% of its mitogenome.

CENSOR [41] was used to identify integrated transposable elements (TEs) in mitogenomes. No complete TEs were detected; however, a total of 33 footprints of these elements were identified in the commercial strain dkN001 (12,061 bp in total), comprising partial sequences of DNA transposons (15.87% of total TE content) and LTR retrotransposons (84.13%). In the wild isolates, 40 elements (15,809 bp) were detected in dkN009 and 36 (16,896 bp) in dkF515. When wild isolates were compared, we observed that the dkN009 strain exhibited a higher proportion of LTR retrotransposon footprints (91.11%) than dkF515 (76.18%). Most of these sequences matched *Ascomycota* fungal entries in the Repbase library of repetitive DNA elements [42] (Table S6).

These findings suggest that repetitive sequences are common features of *P. ostreatus* mitogenomes, particularly TEs, which account for 18.47% of the total mitogenome length in dkN001, 21.63% in dkN009, and 23.3% in dkF515. Such elements could contribute to gene rearrangements, size variation, and intron dynamics in these mitogenomes.

### 3.3. Dynamics of Introns and Intronic ORFs in cox1 and rnl Genes of P. ostreatus Strains

Further analysis revealed that the *rnl* and the *cox1* genes contained intronic sequences harbouring ORFs encoding homing endonucleases (HEs) of the LAGLIDADG and GIY-YIG families.

Six introns were found in the *cox1* gene in the commercial strain dkN001, while nine and ten introns were found in the *cox1* and *rnl* genes in the wild isolates dkF515 and dkN009, respectively. Analysis of the *rnl* gene in the wild isolates revealed the presence of two intronic regions, one of which contains an ORF encoding a LAGLIDADG-type HE. In contrast, the *rnl* gene in the commercial strain dkN001 had lost both intronic regions (Figure 1).

Comparative analysis of the *cox1* gene among strains revealed marked variability in length, as well as in the number and size of exons and introns of this gene. In the commercial dkN001 strain, this gene exhibited the smallest size (9715 bp), followed by the wild isolates dkF515 (11,699 bp) and dkN009 (12,655 bp). In addition, consistent with analysis in other fungal species, the *cox1* genes of the *P. ostreatus* strains in this study utilise GTG as the start codon.

**Figure 2:**
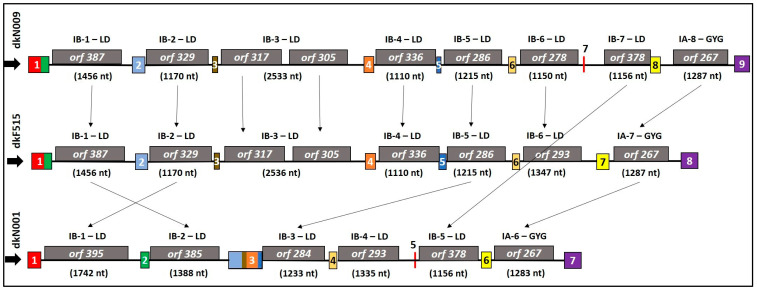
Schematic representation of intron–exon organisation of the *cox1* genes in *P. ostreatus* strains dkN001, dkF515, and dkN009. Coloured boxes represent exons drawn to scale, and identical colours indicate similar amino acid sequences. Grey boxes denote predicted ORFs encoding HEGs of the LAGLIDADG (LD) and GIY-YIG (GIY) families, as identified by the Mfannot tool. The name and size (bp) of each intronic region are indicated between exons. Arrows represent homologous HEGs with >95% sequence similarity.

With respect to exon–intron organisation, the commercial strain dkN001 contained seven exons and six introns, each harbouring an ORF. The wild isolate dkF515 presented eight exons and seven introns containing eight ORFs (one intron contains two ORFs), whereas the other wild isolate, dkN009, exhibited nine exons and eight introns with nine ORFs. As shown in Figure 2 and Figure S2, the observed variations in exon and intron number and size are attributable to intron gain or loss events. Exon lengths ranged from 18 bp (exon 5) to 585 bp (exon 3) in strain dkN001; from 71 bp (exon 5) to 380 bp (exon 1) in dkF515; and from 18 bp (exon 7) to 380 bp (exon 1) in dkN009. Loss of introns resulted in the fusion of adjacent exons, thereby altering the exon–intron organisation. In the commercial strain dkN001, the loss of several introns led to the formation of an elongated exon 3 (585 bp), generated by the fusion of exons 2, 3, 4, and 5, which remained separate in the wild isolates dkF515 and dkN009. Conversely, the commercial strain dkN001 exhibited an intron insertion event between exons 1 and 2, which was absent in both wild isolates. Therefore, the commercial strain dkN001 has not only undergone intron loss, resulting in a considerably smaller in gene size, but this reduction has also been partly counteracted by intron proliferation. Although exon lengths varied among strains, the total length of the *cox1* gene ORF was highly conserved with 1578 bp across all strains. Therefore, the size differences observed in the *cox1* gene among strains can be attributable exclusively to intron gain or loss events.

The small size of the exons contrasts with the large size of the introns observed in all strains (Table 1). Intron lengths ranged from 1109 bp (intron 4 in dkN009 and dkF515) to 2536 bp (intron 3 in dkF515), with an average length of 1400 bp. Introns accounted for over 86% of the total *cox1* gene length in wild isolates, but only 83.7% in dkN001, consistent with its smaller gene size. This reduction in the commercial strain may reflect genomic rearrangements linked to domestication and prolonged subculturing under laboratory conditions.

All introns identified in the *cox1* genes belonged to group I and contained ORFs encoding for single- or double-motif LAGLIDADG (LD type IB) or GIY-YIG (GIY type IA). Group I introns were located at six insertion sites in strain dkN001 (six HEs), seven in dkF515 (eight HEs), and eight in dkN009 (nine HEs). The number of HEs and the cumulative length of ORFs encoding endonucleases showed a strong correlation with the size of the *cox1* gene (R^2^ = 0.99). Interestingly, the *cox1* gene of the wild isolates was the only one containing two LAGLIDADG-type HEs in the same intron (intron 3).

All strains harboured a GIY-YIG endonuclease in the last intron of the gene. HE analysis revealed high similarity in both copy number and protein identity between dkN009 and dkF515, with 99–100% identity between HEs of introns 1 to 5 (Figure 2). In contrast, commercial strain dkN001 displayed marked differences in both HE number and sequence identity, mainly due to the presence of a novel LD in intron 1 and the absence of introns 2, 3, and 4 observed in the wild isolates, resulting in a considerable reduction in cox1 gene size (Figure S2). Most LDs identified in the intronic regions of the *cox1* genes contained the conserved LAGLIDADG_1 (Pfam00961) motif and contained two conserved acidic residues (D or E) required for metal-ion binding and catalysis (Figure S3). Notably, the LD of *orf329* (intron 2) and *orf336* (intron 4) in strain dkN009, as well as the LD of *orf336* (intron 4) in strain dkF515, lacks one of these residues. The sequences of the LAGLIDADG_1 motifs identified in the HEGs of the *cox1* gene showed high variability both inside and among strains.

Taken together, the comparisons among mitogenomes indicate that the wild isolates (dkN009 and dkF515) share greater similarity with each other than with the commercial strain (dkN001), highlighting a clear genetic distinction between wild and commercial lineages.

### 3.4. Effects of Environmental Stressors on Growth and Mitochondrial Gene Expression

Given that mitochondria constitute the primary energy-producing organelles of the cell, the phenotype, measured as growth rate, together with the relative expression profiles of mitochondrial PCGs, was examined in each strain grown under a range of stress conditions, including temperature shifts and alternative carbon sources. These analyses aimed to characterise the phenotypic plasticity of each strain, as inferred from the growth and mitochondrial expression levels of PCGs, in response to environmental perturbations.

We examined the differential expression of mitochondrial genes from two complementary perspectives: (i) intra-strain differences under different culture conditions and (ii) inter-strain differences within the same culture condition.

#### 3.4.1. Temperature Effects on Growth Rate and Mitochondrial Gene Transcription Levels

Dikaryotic strains were grown at 15 °C (low-temperature stress) and 32 °C (high-temperature stress) on MESM, as well as SC (24 °C), and both their growth rates and the expression profiles of 14 PCGs by RT-qPCR experiments were analysed. We examined seven genes encoding proteins of the NADH dehydrogenase complex I (*nad1*, *nad2*, *nad3*, *nad4*, *nad4L*, *nad5* and *nad6*), one gene of cytochrome bc1 complex III (*cob*), three genes of cytochrome c oxidase complex IV (*cox1*, *cox2* and *cox3*), and three genes of ATP synthase complex V (*atp6*, *atp8* and *atp9*).

An overview of the expression level of the mitochondrial genes analysed under these growth conditions revealed that, in all the strains, the mitochondrial genes encoding the NADH dehydrogenase complex I exhibited the lowest expression, whereas ATP synthase subunit 9 (*atp9*) of complex V and the cytochrome c oxidase subunit 1 (*cox1*) of complex IV showed the highest (Figure S4).

When we analysed the intra-strain growth rate differences at different temperatures (Figure S5A), we could observe that the commercial dkN001 strain and the wild isolate adapted to laboratory conditions dkF515 (Figure S5C) exhibited similar patterns, with the highest values observed at 32 °C, whereas the wild isolate dkN009 (Figure S5B) showed higher growth rates at 32 °C and under SC compared with those at 15 °C.

**Figure 3:**
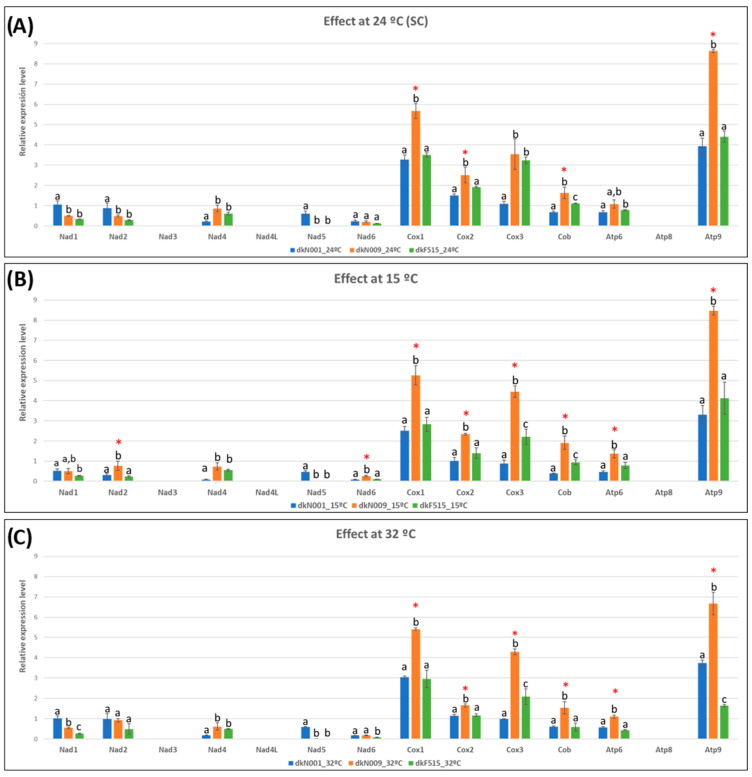
Expression levels of mitochondrial PCGs in the dikaryotic strains dkN001, dkN009, and dkF515 grown in MESM (**A**) under (24 °C), (**B**) at 15 °C, and (**C**) at 32 °C. Red asterisks indicate upregulation of mitochondrial PCGs of wild-type strain dkN009. Different lower letters indicate, in each analysis, significant differences at level of *p*-value < 0.05 according to Scheffe’s test.

When we studied gene expression, we observed that in the commercial strain dkN001 (Figure S6A), the *cox1* and *atp9* genes displayed the highest expression levels. Significant differential expression was detected in genes of complex I (*nad1*, *nad2*, *nad4),* complex III (*cob*), and complex IV (*cox1*) at 32 °C and under SC compared to 15 °C, as well as in *cox2* under SC compared with the remaining conditions. In contrast, the wild isolate dkN009 (Figure S6B) showed similar expression levels across all mitochondrial PCGs at different temperatures, except for *cox2* and *atp9*, whose expression was lower at 32 °C than at 15 °C and SC (Fold Change < 1.31). The wild isolate adapted to laboratory conditions, dkF515 (Figure S6C), exhibited higher expression under SC in complex IV genes (*cox2*, *cox3*) compared with the other conditions (FC > 1.4), and increased expression at 15 °C and SC in complex III and V genes, with FC > 1.36 for *cob*, >1.2 for *atp6*, and >2.6 for *atp9* genes. Moreover, genes encoding proteins of complexes III, IV and V exhibited significantly higher expression levels in the wild isolate dkN009 compared with both strains adapted to laboratory culture conditions (dkN001 and dkF515) (Figure 3, Figures S4 and S6). Particularly, high fold changes were observed for the *cox1* gene (FC > 1.8), *cox3* (FC > 2.5 relative to dkN001 and FC > 1.6 relative to dkF515) and *atp9* gene (FC > 2.4).

When examining growth rate differences among strains under different temperatures, the wild isolate dkN009 displayed reduced rates under high- and low-temperature stress (approximately 15–22%) (Figure S5D–F). Also, the analysis revealed upregulation of most PCGs in dkN009 compared with the dkN001 and dkF515 strains across all temperature conditions (Figure 3A–C), except for the mitochondrial genes encoding complex I.

Taken together, these findings suggest that the two strains adapted to laboratory culture conditions (dkN001 and dkF515) exhibit comparable expression profiles of PCGs across temperatures and respond in a similar manner, in contrast to the distinct profile observed in the wild isolate dkN009, despite the fact that the mitogenomes of the two wild isolates (dkF515 and dkN009) share more than 99% sequence identity.

#### 3.4.2. Carbon Sources Effects on Growth Rate and Mitochondrial Gene Transcription Levels

We analysed both growth rate and the expression levels of mitochondrial PCGs in *P. ostreatus* strains grown under three different carbon source conditions: SC (MESM medium), MSM supplemented with 1% sucrose, and MSM supplemented with 1% glycerol.

We observed that the growth rate of all strains was markedly affected by MSM supplemented with glycerol, resulting in a notable reduction in growth across all strains. This effect was significantly more pronounced in the commercial strain dkN001, which showed a 32.2% reduction relative to SC and a 47.5% reduction relative to MSM supplemented with sucrose (Figure S7).

**Figure 4:**
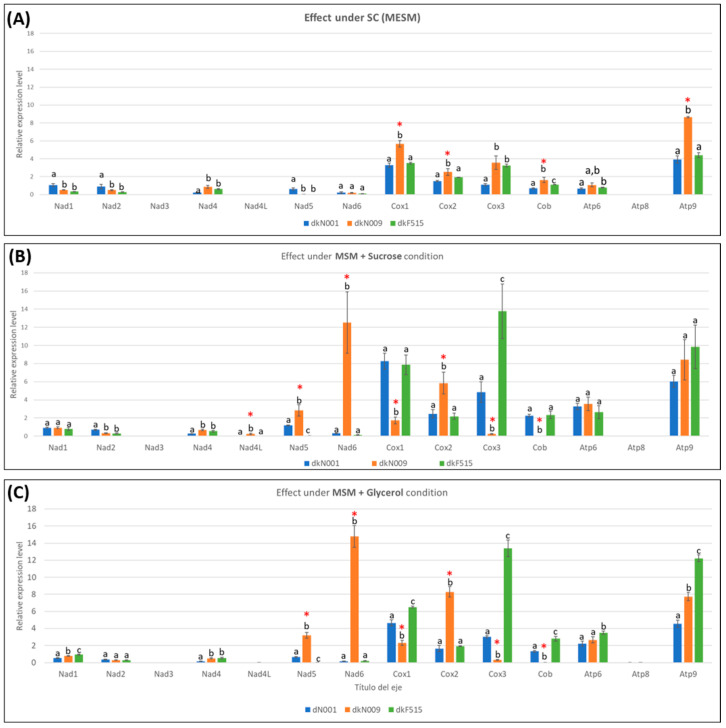
Expression levels of mitochondrial PCGs in the dikaryotic strains dkN001, dkN009 and dkF515 grown in (**A**) SC (MESM), (**B**) MSM supplemented with 1% of sucrose, and (**C**) MSM supplemented with 1% glycerol. Red asterisks indicate up- or downregulation of mitochondrial PCGs of wild-type strain dkN009. Different lower letters indicate, in each analysis, significant differences at level of *p*-value < 0.05 according to Scheffe’s test.

Analysis of mitochondrial gene transcription revealed that, for most genes, the expression profiles under different carbon sources resembled those observed under different temperature conditions. A notable exception was the *nad6* gene of complex I in the wild isolate dkN009, which displayed expression levels comparable to the most highly expressed mitochondrial genes, with FC > 65 relative to dkN009 grown under SC and to dkN001 or dkF515 grown under all conditions (Figure S8). In addition, opposite expression patterns were observed when strains were grown in SC compared to MSM supplemented with sucrose or with glycerol. For instance, the *cox1* gene exhibited higher expression levels in the laboratory-adapted strains dkN001 and dkF515 when grown in minimal medium, whereas lower expression was detected in the wild isolate dkN009 under the same conditions. In contrast, under SC conditions, the highest *cox1* expression was observed in dkN009 compared with the other strains (Figure 4).

When examining intra- and inter-strain variations in mitochondrial transcriptional profiles under different carbon source conditions, we found that the strains dkN001 and dkF515, both adapted to laboratory culture conditions, displayed markedly elevated expression of mitochondrial PCGs associated with complexes III (*cob*) and IV (*cox1* and *cox3*) when they were cultured in MSM supplemented with sucrose (FC > 2.2 for *cox1*; FC > 4.2 for *cox3*; FC > 3.28 and >2.1 for *cob* in dkN001 and dkF515, respectively) or glycerol (FC > 1.5 for *cox1*; FC > 2.7 and >4.1 for *cox3* in dkN001 and dkF515, respectively; FC > 1.7 for *cob*), relative to those grown in SC. Therefore, the wild isolate dkN009 exhibited reduced expression of these genes, but showed increased expression of the complex I genes *nad5* and *nad6*, as well as the complex IV gene *cox2*, under the same sucrose or glycerol conditions (Figure 4 and Figure S9).

Collectively, these findings indicate that the dkN001 and dkF515 strains adapted to laboratory conditions share a conserved mitochondrial gene expression profile across different carbon sources, which diverges substantially from that of the wild isolate dkN009. These results suggest that the long-term laboratory maintenance has led to altered regulation of mitochondrial gene expression, ultimately contributing to laboratory domestication.

## 4. Discussion

Mitochondria are central to cellular energy metabolism and play a key role in fungal growth, stress responses, and adaptation. Fungal mitochondrial genomes are particularly dynamic, exhibiting extensive variation in size, structure, and intron content across taxa [12]. In this study, we analysed the complete mitochondrial genomes and mitochondrial gene expression profiles of three dikaryotic strains of *Pleurotus ostreatus* differing in origin and degree of laboratory adaptation. Our results provide integrated structural and functional evidence that long-term laboratory domestication is associated with mitochondrial genome streamlining and coordinated changes in mitochondrial gene regulation.

Previous studies have shown that mitochondrial genome size in fungi is highly variable, often exhibiting extensive gene rearrangements [12]. Among basidiomycetes, *Elmerina hispida* MES06 possesses the largest mitochondrial genome sequenced to date, with 265,604 bp (GenBank accession: NC_086750), whereas *Cryptococcus neoformans* var. *grubii* strain TW1 has the smallest known mitochondrial genome, with 24,864 bp (GenBank accession: CM054775). In this study, the complete nucleotide sequences of the mitochondrial genomes were established for three strains of *P. ostreatus*, two wild strains (dkN009 and dkF515) and one commercial strain (dkN001). Analysis of the mitochondrial genome of the *P. ostreatus* dikaryotic strains revealed that the mitogenome of the commercial dkN001 strain, with 65,303 bp, is one of the smallest mitochondrial genomes reported within the genus *Pleurotus*, larger only than that of *P. citrinopileatus* (60,694 bp; NC_036998) and followed by *P. pulmonarius* strain ZA3 (68,305 bp; MH460534), *P. pulmonarius* (70,671 bp; NC_061177); dkF515 *P. ostreatus* strain of this study (72,132 bp); *P. eryngii* (72,650 bp; KX827267), dkN009 *P. ostreatus* strain analysed in this work (73,085 bp); *P. ostreatus* strain P51 (73,242 bp; EF204913), *P. platypus* (73,807 bp; MG017445), *P. pulmonarius* strain X1–15 (73,435 bp; MG579954), and *P. giganteus* (102,950 bp; OM681506) [12,15,43,44]. The observed differences in size and sequence similarity among the three dikaryotic strains of this study suggest an evolutionary divergence linked to the domestication or artificial selection of dkN001, consistent with patterns reported in domesticated *Agaricus bisporus* strains [45].

Variations in fungal mitochondrial genome size are generally attributed mainly to the accumulation of duplicated repeats, horizontal gene transfer, and differences in intron content [12]. Our findings demonstrate size polymorphism between commercial and wild isolates of *P. ostreatus*, with the mitogenome of the commercial strain being approximately 10% smaller than that of their wild counterparts. Mitogenomes of this study contain numerous repetitive elements, representing mobile sequences capable of increasing genome size, as previously reported in other fungal mitogenomes such as *Ophiocordyceps sinensis* [46], *Rhizoctonia solani* [47], and yeast [48], among others. However, no differences in the abundance of repetitive elements were detected between commercial and wild strains, suggesting that the accumulation of such elements is not the primary factor underlying their size variation. In this sense, different studies have shown that some yeasts used in the food and biotechnology industries (such as *S. cerevisiae*, industrial yeasts, and some filamentous fungi) exhibit rearrangements in their mitochondrial genomes that include the loss or gain of introns and other mobile elements. Specially, some strains of *S. cerevisiae* used in the production of bread, wine and beer have been found to possess mitochondrial genomes with significant variations from their wild relatives. These industrial strains, which have fewer introns in their mitogenomes, show better performance in fermentation processes. These variations include the loss of introns and the presence of mobile elements which could facilitate their adaptation to the artificial environments where they grow up [49]. In filamentous fungi, it has been found that the loss of introns in mitochondrial genes can be correlated with adaptations to specific environments. Domesticated fungi, or those subjected to controlled cultivation, often show simpler mitochondrial genomes [45]. Our findings confirm that the commercial strain dkN001, cultivated under controlled conditions, exhibits a reduced mitochondrial genome relative to the wild isolates including rearrangements of mitochondrial PCGs. These rearrangements have been previously observed between domesticated and wild fungal strains, including *A. bisporus*, *Agrocybe aegerita*, and *S. cerevisiae* [45,49,50], as well as species of the genus *Pleurotus*, such as *P. citrinopileatus*, *P. eryngii,* and *P. pulmonarius* [12,15,51].

When the sizes of mitochondrial genomes of different basidiomycetes are compared and associated with their lifestyles, a trend toward smaller sizes in biotroph/parasite fungi compared to saprotrophs can be observed. In this scenario, laboratory subculturing of wild strains could trigger a similar process to that observed in parasitism/biotrophy.

Even though the gene order is highly conserved among the *P. ostreatus* strains analysed in this study, we could observe an inverted region of the nine kilobases approximately affecting two genes of complex I (*nad2* and *nad3*), one gene of complex V (*atp9*), the small subunit ribosomal RNA (*rns*), as well as eight tRNAs. It is known that the rearrangements not only involve mitochondrial PCGs but also influence the organisation of introns within those genes where they occur [52]. Introns are regarded as mobile genetic elements within eukaryotic mitochondrial genomes, and their dynamic variation exerts a substantial effect on mitogenome organisation [53,54]. When we analysed the contribution of intergenic and intronic regions to the total mitogenome size of *P. ostreatus* strains dkN001, dkF515, and dkN009 (Figure 1 and Figure S10), we found that the intergenic regions grouping intergenic and intergenic hypothetical protein-coding (ncORFs) sequences accounted for 50.2%, 49.1%, and 48% of the total mitogenome length, respectively. In contrast, the intronic regions comprised 8137 bp, 11,888 bp, and 12,841 bp, corresponding to 12. 5% in the commercial strain dkN001 and 16.5% and 17.6% in the wild isolates dkF515 and dkN009, respectively. This is consistent with previous reports showing that intron and intronic regions have a significant influence on the broad variation in fungal mitogenome size [55]. The introns identified in the *P. ostreatus* strains dkN001, dkF515, and dkN009 were found to correspond to group I introns. This class of introns occurs in a range of genetic contexts, including rRNA, tRNA, and mRNA genes within organelles of fungi, plants, and protists [56]. Group I introns are catalytic RNAs capable of self-splicing both in vitro and in vivo [57,58]. In fungi, these intronic regions also harbour ORFs that commonly encode proteins with homing endonuclease (HE) functions and have been associated with mitogenome size variation [45,52,59]. When we analysed intron regions, we observed that the commercial strain dkN001 contained introns exclusively within the *cox1* gene, whereas the wild isolates dkF515 and dkN009 harboured introns in both the *cox1* and *rnl* genes. Similar patterns have been reported in *P. pulmonarius*, where the mitogenomes of some strains contain introns solely in the *cox1* gene [51], while other strains of genus *Pleurotus* harbour intronic regions in both the *cox1* and *rnl* genes [12]. Our comparative analysis of the *cox1* gene revealed substantial variation in total gene length among *P. ostreatus* strains. Differences in the distribution of exons along the *cox1* gene were observed, although these did not affect the length of the open reading frame (ORF). Therefore, variation in gene size was attributable exclusively to differences in the number and length of intronic regions. In this study, the commercial strain dkN001 exhibited a loss of introns within the *cox1* gene, a feature also reported in domesticated strains of other basidiomycete species within the genus *Agaricus* [45,60]. This supports our hypothesis that intronic HEs might be involved in the shrinking of mitogenome size during the domestication of dkN001 strain.

The intron dynamics observed as the gain and loss of these regions in the mitochondrial genome have been associated with mechanisms regulating gene expression in response to controlled environmental conditions. Mitochondrial introns located in genes such as *cox1*, *cob* and *rnl* are not merely neutral elements but can influence splicing efficiency, transcript processing, and mRNA stability, thereby affecting mitochondrial transcriptional homeostasis and respiratory function, as was observed in the *nde1* (complex I) gene expression in *P. ostreatus* [25]. Intron removal has been shown to disrupt normal expression of *cox1* and other respiratory genes by affecting mRNA stability and transcript abundance. Intron-less mitochondrial genomes can accumulate excess mature transcripts altering the stoichiometry of respiratory complexes and leading to physiological stress, as demonstrated in *S. cerevisiae* [61]. It is important to note that the effects of mitochondrial (prokaryotic-type) intron processing differ from those observed in nuclear intron processing.

Previous studies on the ascomycete *Podospora anserina*, a fungus widely used in research on ageing and mitochondrial biology, revealed a complex mitochondrial genomic structure containing mobile introns that contribute to the regulation of cellular processes involved in adaptation to laboratory conditions [62]. These findings support the hypothesis that the loss of mobile introns—such as those encoding LAGLIDADG endonucleases observed in the *rnl* and *cox1* genes—may reflect a process of mitochondrial genome domestication. In this context, selective pressures under controlled conditions may favour increased genomic stability and replication efficiency, leading to the elimination of intronic regions that no longer confer adaptive advantages [55,63]. Similarly, other commercial and domesticated mushrooms, such as *Agaricus bisporus*, have exhibited restricted intron distribution patterns within their mitochondrial genomes, frequently displaying intron loss compared with wild strains. This trend suggests selection for a simplified mitogenome during cultivation [45]. Therefore, domestication may promote intron loss or mitochondrial genome streamlining, reflecting adaptation to stable cultivation.

Adaptation may proceed via natural selection acting on genetic variation, thereby conferring greater fitness to individuals under specific environmental regimes, referred to as ‘adaptive evolution’. In such cases, it may be possible to identify genes that play a key role in this process of adaptive evolution. However, the adaptation may also occur through phenotypic plasticity, which does not arise from molecular evolution but rather represents a physiological response to environmental changes [64,65].

It has been reported that the mitonuclear interactions can affect growth rate, as occurs in both Ascomycota and Basidiomycota fungi [25,66,67,68]. So, mitonuclear interactions are essential to the functioning of mitochondria in eukaryotic cells, including the production of ATP for cellular energy, and mitochondrial dysfunction resulting from these incompatibilities is frequently linked to detrimental fitness. The protein components of the electron transport system show the most well-characterised instances of mitonuclear incompatibility [25,67,68,69].

Due to phenotypic evolution being closely associated with alterations in gene expression [29], we investigated the potential genetic mechanisms underlying the adaptation of wild isolates to laboratory culture conditions by qPCR analyses. It has been observed by transcriptomic analyses of domesticated species such as the rat, rainbow trout, rice, chicken or maize that domestication markedly alters gene expression profiles and generally reduces overall expression diversity relative to wild counterparts [29,70]. Consequently, domestication tends to reshape energy metabolism at the level of gene expression. In plants, phenotypic plasticity of traits is frequently assessed to enhance understanding of organismal and ecosystem responses to climate change. In fungi, the domestication of edible mushrooms such as *Flammulina filiformis* shows genomic and transcriptomic variations between cultivated and wild populations [71].

Consistent with patterns observed in domesticated animals, plants or fungi, where gene expression and mitochondrial genome evolution diverge significantly from wild ancestors, our findings suggest that, in *P. ostreatus,* domestication influences both the intron architecture of mitochondrial protein-coding genes such as *cox1* and *rnl* and the differential expression of specific mitochondrial PCGs. This supports the hypothesis that domestication has influenced both nuclear gene regulatory networks and mitochondrial function, likely as a result of adaptation to artificial culture environments compared with the wild.

Furthermore, the longer the culture time under control domesticated conditions, the deeper the modification observed, as shown when the laboratory-domesticated strain for nearly 30 years, dkN001, is compared with dkN009, an essentially wild strain. In this sense, our results indicate that adaptation to laboratory conditions of dkN001 and dkF515 strains has been accompanied by substantial modifications in the energy metabolism manifested through altered gene expression levels in comparison with those of the wild isolate dkN009. In this study, we could observe that domestication/adaptation of *Pleurotus* strains could be associated with changes in the expression profiles of mitochondrial PCGs corresponding to complexes III, IV, and V. Several studies support the distinction between the concepts of domestication and adaptation to laboratory conditions. In *S. cerevisiae*, for example, strains maintained under laboratory conditions over thousands of generations show predictable adaptations in growth rate and metabolic regulation that are not necessarily associated with deliberate selection for industrial traits [72]. Conversely, domesticated yeasts used in brewing, baking or winemaking exhibit signatures of selection on metabolic pathways linked to fermentation performance, stress tolerance, and flavour compound production, reflecting human-mediated selection rather than neutral laboratory adaptation [73]. In summary, while domestication refers to human-driven selection for desirable commercial traits, adaptation broadly refers to selection processes occurring in a neutral or stable environment, highlighting the importance of distinguishing between these two concepts in fungal biology.

Our findings suggest that the mitochondrial response to environmental variations, inferred from the expression analysis of mitochondrial PEGs, is not solely a consequence of strain-specific phenotypic plasticity manifested as intra-strain differences under varying culture conditions, but is also influenced by mitonuclear interactions observed in different *P. ostreatus* strains [25]. This is supported by the differential expression of mitochondrial PCGs observed between the wild isolates dkF515 and dkN009, despite their mitogenomes sharing over 99% sequence identity. The observed differential expression patterns in the strains likely reflect a process of evolutionary adaptation to laboratory culture conditions in the commercial strain dkN001 and the wild isolate dkF515, indicating that pronounced alterations in energy metabolism, manifested as shifts in the mitochondrial expression profiles, accompany the domestication/adaptation process. This process reflects the adaptation of a wild isolate to laboratory culture conditions, while leaving its genomic organisation largely unaltered, as has been observed in other organisms such as animals and plants [29,39,70]. Further validation in larger strain collections will be necessary to confirm the consistency of differential expression patterns of PCGs associated with domestication-driven changes.

Taken together, our results suggest that domestication in *P. ostreatus* involves two complementary processes: (i) structural streamlining of the mitochondrial genome through intron loss and genome reorganisation, and (ii) functional reprogramming of mitochondrial gene expression linked to altered energy demands under controlled culture conditions. These changes appear to be driven by long-term laboratory maintenance rather than by strain origin alone, as evidenced by the convergence of mitochondrial expression profiles in independently derived laboratory-adapted strains.

## Figures and Tables

**Table 1:** Intron types and intronic open reading frames identified in the mitochondrial *cox1* gene of *P. ostreatus* strains dkN001, dkF515 and dkN009.

Strains	Gene	Intron Number	Intron Class ^a^	Intron Length (bp)	Intronic ORF	Conserved Domain of HEGs	Number of Repeat Motifs (HEs)	Intronic Region in *cox1* Gene (bp)	Coding Sequence (CDS) Region	Fisrt Amino Acid (CDS)
*dkN001*	*cox1*	I1	IB	1742	*orf395*	LAGLIDADG_1	2	235–1976	235–1422	K (Lysine)
	*cox1*	I2	IB	1391	*orf385*	LAGLIDADG_1	1	2123–3510	2124–3281	L (Leucine)
	*cox1*	I3	IB	1233	*orf284*	LAGLIDADG_2	1	4096–5328	4097–4951	S (Serine)
	*cox1*	I4	IB	1335	*orf293*	LAGLIDADG_1	2	5465–6799	5465–6346	R (Arginine)
	*cox1*	I5	IB	1156	*orf378*	LAGLIDADG_1	1	6818–7973	6818–7954	Q (Glutamine)
	*cox1*	I6	IA	1283	*orf267*	GIY-YIG	1	8154–9436	8154–8957	V (Valine)
*dkF515*	*cox1*	I1	IB	1456	orf387	LAGLIDADG_1	2	381–1836	382–1545	L (Leucine)
	*cox1*	I2	IB	1170	orf329	LAGLIDADG_1	2	2066–3235	2066–3055	K (Lysine)
	*cox1*	I3	IB	2536	orf317	LAGLIDADG_1	2	3341–5876	3341–4294	I (Isoleucine)
	*cox1*	I3	IB	2536	orf305	LAGLIDADG_1	1	3341–5876	4882–5799	M (Methionine)
	*cox1*	I4	IB	1110	orf336	LAGLIDADG_1	2	6057–7166	6057–7067	R (Arginine)
	*cox1*	I5	IB	1215	orf286	LAGLIDADG_2	1	7238–8452	7239–8099	S (Serine)
	*cox1*	I6	IB	1347	orf293	LAGLIDADG_1	1	8589–9935	8589–9470	R (Arginine)
	*cox1*	I7	IA	1287	orf267	GIY-YIG	1	10,134–11,420	10,134–10,937	V (Valine)
*dkN009*	*cox1*	I1	IB	1456	orf387	LAGLIDADG_1	2	381–1836	382–1545	L (Leucine)
	*cox1*	I2	IB	1170	orf329	LAGLIDADG_1	2	2066–3235	2066–3055	K (Lysine)
	*cox1*	I3	IB	2533	orf317	LAGLIDADG_1	2	3341–5873	3341–4294	I (Isoleucine)
	*cox1*	I3	IB	2533	orf305	LAGLIDADG_1	1	3341–5873	4880–5797	M (Methionine)
	*cox1*	I4	IB	1110	orf336	LAGLIDADG_1	2	6054–7163	6054–7064	R (Arginine)
	*cox1*	I5	IB	1215	orf286	LAGLIDADG_2	1	7235–8449	7236–8096	S (Serine)
	*cox1*	I6	IB	1150	orf278	LAGLIDADG_1	2	8586–9735	8586–9422	T (Threonine)
	*cox1*	I7	IB	1156	orf378	LAGLIDADG_1	1	9754–10,909	9754–10,890	Q (Glutamine)
	*cox1*	I8	IA	1287	orf267	GIY-YIG	1	11,090–12,376	11,090–11,893	V (Valine)

^a^ Intron class determined by using Mfannot tool. IB correspond to HEs with LAGLIDADG conserved domain and IA correspond to HEs with GYY_YIG conserved domain.

## Data Availability

Mitogenomes sequences are available in the NCBI database. The data presented in this study are available on request from the corresponding author.

## Supplementary Materials

The following supporting information can be downloaded at: https://www.mdpi.com/article/10.3390/jof12010075/s1, Figure S1: Predicted secondary structures of all tRNAs identified in the mitogenome of the dkN001 strain are shown. For the dkN009 and dkF515 strains, only the tRNA secondary structures that differ from those in dkN001 are presented; Figure S2: Structural alignments of the *cox1* sequences from dkN001, dkF515, and dkN009 based on analyses with the Mauve and MFannot tools; Figure S3: (a) Sequence logo of the putative LAGLIDADG_1 motifs located within introns of *cox1* gene P. ostreatus strains dkN001, dkN009 and dkF515. Conserved residues (D or E) are highlighted with dashed boxes. Information content at each position (in bits; X axis) is represented by the height of the amino acid. A score of 3.2 bits indicates high conservation, whereas a score of 0 implies low conservation. (b) Alignment of the putative LAGLIDAD_1 motifs. Amino acids are colour-coded according to chemical properties (blue, basic; red, acid; green, polar neutral; black hydrophobic); Figure S4: Relative expression of mitochondrial PCGs under SC (24 °C), and low-temperature (15 °C) and high-temperature (32 °C) stresses; Figure S5: Growth rate of dikaryotic strains under SC (24 °C) conditions and at different temperatures; Figure S6: Expression levels of mitochondrial PCGs in (A) dkN001, (B) dkN009, and (C) dkF515 strains at 15, 24 and 32 °C; Figure S7: Growth rate of dikaryotic strains under SC (MESM medium) conditions and with different carbon sources; Figure S8: Relative expression of mitochondrial PCGs under different carbon sources conditions: SC (MESM), MSM supplemented with 1% sucrose and MSM supplemented with 1% glycerol; Figure S9: Expression levels of mitochondrial PCGs in the dikaryotic strains (A) dkN001, (B) dkN009 and (C) dkF515 grown under different carbon sources; Figure S10: Proportion (%) of different components in the mitogenomes of dkN001, dkF515, and dkN009 strains of *P. ostreatus*; Table S1: List of primers; Table S2: Data of genome sequencing; Table S3: Condon usage of tRNAs; Table S4: Duplicated sequences intra mitogenomes; Table S5: Tandem repeats of mitogenomes; Table S6: Transposon elements.

## References

[1] Osellame L.D., Blacker T.S., Duchen M.R. (2012). Cellular and Molecular Mechanisms of Mitochondrial Function. Best. Pract. Res. Clin. Endocrinol. Metab..

[2] Kulik T., Van Diepeningen A.D., Hausner G. (2020). Editorial: The Significance of Mitogenomics in Mycology. Front. Microbiol..

[3] Kurland C.G., Andersson S.G.E. (2000). Origin and Evolution of the Mitochondrial Proteome. Microbiol. Mol. Biol. Rev..

[4] Poyton R.O., McEwen J.E. (1996). Crosstalk Between Nuclear and Mitochondrial Genomes. Annu. Rev. Biochem..

[5] Mattila H., Österman-Udd J., Mali T., Lundell T. (2022). Basidiomycota Fungi and ROS: Genomic Perspective on Key Enzymes Involved in Generation and Mitigation of Reactive Oxygen Species. Front. Fungal Biol..

[6] Olson Å., Stenlid J. (2001). Mitochondrial Control of Fungal Hybrid Virulence. Nature.

[7] Zhang Y., Wang S., Li H., Liu C., Mi F., Wang R., Mo M., Xu J. (2021). Evidence for Persistent Heteroplasmy and Ancient Recombination in the Mitochondrial Genomes of the Edible Yellow Chanterelles from Southwestern China and Europe. Front. Microbiol..

[8] Verma S., Shakya V.P.S., Idnurm A. (2018). Exploring and Exploiting the Connection between Mitochondria and the Virulence of Human Pathogenic Fungi. Virulence.

[9] Lavín J.L., Oguiza J.A., Ramírez L., Pisabarro A.G. (2008). Comparative Genomics of the Oxidative Phosphorylation System in Fungi. Fungal Genet. Biol..

[10] Malina C., Larsson C., Nielsen J. (2018). Yeast Mitochondria: An Overview of Mitochondrial Biology and the Potential of Mitochondrial Systems Biology. FEMS Yeast Res..

[11] Li Q., Yang L., Xiang D., Wan Y., Wu Q., Huang W., Zhao G. (2020). The Complete Mitochondrial Genomes of Two Model Ectomycorrhizal Fungi (*Laccaria*): Features, Intron Dynamics and Phylogenetic Implications. Int. J. Biol. Macromol..

[12] Li Q., Chen C., Xiong C., Jin X., Chen Z., Huang W. (2018). Comparative Mitogenomics Reveals Large-Scale Gene Rearrangements in the Mitochondrial Genome of Two *Pleurotus* Species. Appl. Microbiol. Biotechnol..

[13] Megarioti A.H., Kouvelis V.N. (2020). The Coevolution of Fungal Mitochondrial Introns and Their Homing Endonucleases (GIY-YIG and LAGLIDADG). Genome Biol. Evol..

[14] Férandon C., Moukha S., Callac P., Benedetto J.-P., Castroviejo M., Barroso G. (2010). The *Agaricus bisporus* Cox1 Gene: The Longest Mitochondrial Gene and the Largest Reservoir of Mitochondrial Group I Introns. PLoS ONE.

[15] Ye L.-Y., Deng Y.-J., Mukhtar I., Meng G.-L., Song Y.-J., Cheng B., Hao J.-B., Wu X.-P. (2020). Mitochondrial Genome and Diverse Inheritance Patterns in *Pleurotus pulmonarius*. J. Microbiol..

[16] Wang X., Jia L., Wang M., Yang H., Chen M., Li X., Liu H., Li Q., Liu N. (2020). The Complete Mitochondrial Genome of Medicinal Fungus *Taiwanofungus camphoratus* Reveals Gene Rearrangements and Intron Dynamics of Polyporales. Sci. Rep..

[17] Girmay Z., Gorems W., Birhanu G., Zewdie S. (2016). Growth and Yield Performance of *Pleurotus ostreatus* (Jacq. Fr.) Kumm (Oyster Mushroom) on Different Substrates. AMB Express.

[18] Viriato V., Mäkelä M.R., Kowalczyk J.E., Ballarin C.S., Loiola P.P., Andrade M.C.N. (2022). Organic Residues from Agricultural and Forest Companies in Brazil as Useful Substrates for Cultivation of the Edible Mushroom *Pleurotus ostreatus*. Lett. Appl. Microbiol..

[19] Nakazawa T., Kawauchi M., Otsuka Y., Han J., Koshi D., Schiphof K., Ramírez L., Pisabarro A.G., Honda Y. (2024). *Pleurotus ostreatus* as a Model Mushroom in Genetics, Cell Biology, and Material Sciences. Appl. Microbiol. Biotechnol..

[20] Gafforov Y., Yamaç M., İnci Ş., Rapior S., Yarasheva M., Rašeta M., Khojimatov O.K., Gafforov Y., Bussmann R.W. (2023). Pleurotus Eryngii (DC.) Quél.; *Pleurotus ostreatus* (Jacq.) P. Kumm.-PLEUROTACEAE. Ethnobiology of Uzbekistan: Ethnomedicinal Knowledge of Mountain Communities.

[21] Krupodorova T., Barshteyn V., Tsygankova V., Sevindik M., Blume Y. (2024). Strain-Specific Features of *Pleurotus ostreatus* Growth in Vitro and Some of Its Biological Activities. BMC Biotechnol..

[22] Larraya L., Peñas M.M., Pérez G., Santos C., Ritter E., Pisabarro A.G., Ramírez L. (1999). Identification of Incompatibility Alleles and Characterisation of Molecular Markers Genetically Linked to the A Incompatibility Locus in the White Rot Fungus *Pleurotus ostreatus*. Curr. Genet..

[23] Larraya L.M., Pérez G., Iribarren I., Blanco J.A., Alfonso M., Pisabarro A.G., Ramírez L. (2001). Relationship between Monokaryotic Growth Rate and Mating Type in the Edible Basidiomycete *Pleurotus ostreatus*. Appl. Environ. Microbiol..

[24] Pérez G., Lopez-Moya F., Chuina E., Ibañez-Vea M., Garde E., López-Llorca L.V., Pisabarro A.G., Ramírez L. (2021). Strain Degeneration in *Pleurotus ostreatus*: A Genotype Dependent Oxidative Stress Process Which Triggers Oxidative Stress, Cellular Detoxifying and Cell Wall Reshaping Genes. J. Fungi.

[25] Garde E., Pérez G., Jiménez I., Calvo M.I., Pisabarro A.G., Ramírez L. (2025). Asymmetric Mitonuclear Interactions Trigger Transgressive Inheritance and Mitochondria-Dependent Heterosis in Hybrids of the Model System *Pleurotus ostreatus*. IMA Fungus.

[26] Bennett C.F., Latorre-Muro P., Puigserver P. (2022). Mechanisms of Mitochondrial Respiratory Adaptation. Nat. Rev. Mol. Cell Biol..

[27] Garvin M.R., Thorgaard G.H., Narum S.R. (2015). Differential Expression of Genes That Control Respiration Contribute to Thermal Adaptation in Redband Trout (*Oncorhynchus mykiss gairdneri*). Genome Biol. Evol..

[28] Wu X., Zhan L., Storey K.B., Zhang J., Yu D. (2025). Differential Mitochondrial Genome Expression of Four Skink Species Under High-Temperature Stress and Selection Pressure Analyses in Scincidae. Animals.

[29] Zeng L., Ming C., Li Y., Su L.-Y., Su Y.-H., Otecko N.O., Liu H.-Q., Wang M.-S., Yao Y.-G., Li H.-P. (2017). Rapid Evolution of Genes Involved in Learning and Energy Metabolism for Domestication of the Laboratory Rat. Mol. Biol. Evol..

[30] Hahn C., Bachmann L., Chevreux B. (2013). Reconstructing Mitochondrial Genomes Directly from Genomic Next-Generation Sequencing Reads—A Baiting and Iterative Mapping Approach. Nucleic Acids Res..

[31] Lang B.F., Beck N., Prince S., Sarrasin M., Rioux P., Burger G. (2023). Mitochondrial Genome Annotation with MFannot: A Critical Analysis of Gene Identification and Gene Model Prediction. Front. Plant Sci..

[32] Bernt M., Donath A., Jühling F., Externbrink F., Florentz C., Fritzsch G., Pütz J., Middendorf M., Stadler P.F. (2013). MITOS: Improved de Novo Metazoan Mitochondrial Genome Annotation. Mol. Phylogenet Evol..

[33] Lorenz R., Bernhart S.H., Höner zu Siederdissen C., Tafer H., Flamm C., Stadler P.F., Hofacker I.L. (2011). ViennaRNA Package 2.0. Algorithms Mol. Biol..

[34] Greiner S., Lehwark P., Bock R. (2019). Organellar Genome DRAW (OGDRAW) Version 1.3.1: Expanded Toolkit for the Graphical Visualization of Organellar Genomes. Nucleic Acids Res..

[35] Darling A.C.E., Mau B., Blattner F.R., Perna N.T. (2004). Mauve: Multiple Alignment of Conserved Genomic Sequence with Rearrangements. Genome Res..

[36] Crooks G.E., Hon G., Chandonia J.M., Brenner S.E. (2004). WebLogo: A Sequence Logo Generator. Genome Res..

[37] Benson G. (1999). Tandem Repeats Finder: A Program to Analyze DNA Sequences. Nucleic Acids Res..

[38] Livak K.J., Schmittgen T.D. (2001). Analysis of Relative Gene Expression Data Using Real-Time Quantitative PCR and the 2−ΔΔCT Method. Methods.

[39] Hisano H., Tsujimura M., Yoshida H., Terachi T., Sato K. (2016). Mitochondrial Genome Sequences from Wild and Cultivated Barley (*Hordeum vulgare*). BMC Genom..

[40] Li Z.-C., Xie T.-C., Feng X.-L., Wang Z.-X., Lin C., Li G.-M., Li X.-Z., Qi J. (2023). The First Five Mitochondrial Genomes for the Family Nidulariaceae Reveal Novel Gene Rearrangements, Intron Dynamics, and Phylogeny of Agaricales. Int. J. Mol. Sci..

[41] Kohany O., Gentles A.J., Hankus L., Jurka J. (2006). Annotation, Submission and Screening of Repetitive Elements in Repbase: Repbase Submitter and Censor. BMC Bioinform..

[42] Bao W., Kojima K.K., Kohany O. (2015). Repbase Update, a Database of Repetitive Elements in Eukaryotic Genomes. Mob. DNA.

[43] Wang Y., Zeng F., Hon C.C., Zhang Y., Leung F.C.C. (2008). The Mitochondrial Genome of the Basidiomycete Fungus *Pleurotus ostreatus* (Oyster Mushroom). FEMS Microbiol. Lett..

[44] Liu Z., Wu S., Chen X., Zhang W., Zhou S., Wang X. (2022). The Complete Mitochondrial Genome of the Edible Mushroom *Pleurotus giganteus* (Agaricales, *Pleurotus*) and Insights into Its Phylogeny. Mitochondrial DNA B Resour..

[45] Zhang M.-Z., Xu J.-P., Callac P., Chen M.-Y., Wu Q., Wach M., Mata G., Zhao R.-L. (2023). Insight into the Evolutionary and Domesticated History of the Most Widely Cultivated Mushroom *Agaricus bisporus* via Mitogenome Sequences of 361 Global Strains. BMC Genom..

[46] Li Y., Hu X.-D., Yang R.-H., Hsiang T., Wang K., Liang D.-Q., Liang F., Cao D.-M., Zhou F., Wen G. (2015). Complete Mitochondrial Genome of the Medicinal Fungus *Ophiocordyceps sinensis*. Sci. Rep..

[47] Losada L., Pakala S.B., Fedorova N.D., Joardar V., Shabalina S.A., Hostetler J., Pakala S.M., Zafar N., Thomas E., Rodriguez-Carres M. (2014). Mobile Elements and Mitochondrial Genome Expansion in the Soil Fungus and Potato Pathogen *Rhizoctonia solani* AG-3. FEMS Microbiol. Lett..

[48] Bágeľová Poláková S., Lichtner Ž., Szemes T., Smolejová M., Sulo P. (2021). Mitochondrial DNA Duplication, Recombination, and Introgression during Interspecific Hybridization. Sci. Rep..

[49] Freel K.C., Friedrich A., Schacherer J. (2015). Mitochondrial Genome Evolution in Yeasts: An All-Encompassing View. FEMS Yeast Res..

[50] Barroso G., Blesa S., Labarere J. (1995). Wide Distribution of Mitochondrial Genome Rearrangements in Wild Strains of the Cultivated Basidiomycete *Agrocybe aegerita*. Appl. Environ. Microbiol..

[51] Yu Y., Liu T., Wang Y., Liu L., He X., Li J., Martin F.M., Peng W., Tan H. (2024). Comparative Analyses of *Pleurotus pulmonarius* Mitochondrial Genomes Reveal Two Major Lineages of Mini Oyster Mushroom Cultivars. Comput. Struct. Biotechnol. J..

[52] Kolesnikova A.I., Putintseva Y.A., Simonov E.P., Biriukov V.V., Oreshkova N.V., Pavlov I.N., Sharov V.V., Kuzmin D.A., Anderson J.B., Krutovsky K.V. (2019). Mobile Genetic Elements Explain Size Variation in the Mitochondrial Genomes of Four Closely-Related *Armillaria* Species. BMC Genom..

[53] Lambowitz A.M. (1989). Infectious Introns. Cell.

[54] Belfort M., Roberts R.J. (1997). Homing Endonucleases: Keeping the House in Order. Nucleic Acids Res..

[55] Pogoda C.S., Keepers K.G., Nadiadi A.Y., Bailey D.W., Lendemer J.C., Tripp E.A., Kane N.C. (2019). Genome Streamlining via Complete Loss of Introns Has Occurred Multiple Times in Lichenized Fungal Mitochondria. Ecol. Evol..

[56] Mukhopadhyay J., Hausner G. (2021). Organellar Introns in Fungi, Algae, and Plants. Cells.

[57] Stoddard B.L. (2014). Homing Endonucleases from Mobile Group I Introns: Discovery to Genome Engineering. Mob. DNA.

[58] Bonen L., Vogel J. (2001). The Ins and Outs of Group II Introns. Trends Genet..

[59] Chen C., Li Q., Fu R., Wang J., Deng G., Chen X., Lu D. (2021). Comparative Mitochondrial Genome Analysis Reveals Intron Dynamics and Gene Rearrangements in Two *Trametes* Species. Sci. Rep..

[60] De La Bastide P.Y., Sonnenberg A., Van Griensven L., Anderson J.B., Horgen P.A. (1997). Mitochondrial Haplotype Influences Mycelial Growth of *Agaricus bisporus* Heterokaryons. Appl. Environ. Microbiol..

[61] Rudan M., Bou Dib P., Musa M., Kanunnikau M., Sobočanec S., Rueda D., Warnecke T., Kriško A. (2018). Normal Mitochondrial Function in *Saccharomyces cerevisiae* Has Become Dependent on Inefficient Splicing. eLife.

[62] Cummings D.J., McNally K.L., Domenico J.M., Matsuura E.T. (1990). The Complete DNA Sequence of the Mitochondrial Genome of *Podospora anserina*. Curr. Genet..

[63] Lim C.S., Weinstein B.N., Roy S.W., Brown C.M. (2021). Analysis of Fungal Genomes Reveals Commonalities of Intron Gain or Loss and Functions in Intron-Poor Species. Mol. Biol. Evol..

[64] West-Eberhard M.J. (2005). Developmental Plasticity and the Origin of Species Differences. Proc. Natl. Acad. Sci. USA.

[65] Alster C.J., Allison S.D., Johnson N.G., Glassman S.I., Treseder K.K. (2021). Phenotypic Plasticity of Fungal Traits in Response to Moisture and Temperature. ISME Commun..

[66] Paliwal S., Fiumera A.C., Fiumera H.L. (2014). Mitochondrial-Nuclear Epistasis Contributes to Phenotypic Variation and Coadaptation in Natural Isolates of *Saccharomyces cerevisiae*. Genetics.

[67] Nguyen T.H.M., Sondhi S., Ziesel A., Paliwal S., Fiumera H.L. (2020). Mitochondrial-Nuclear Coadaptation Revealed through mtDNA Replacements in *Saccharomyces cerevisiae*. BMC Evol. Biol..

[68] Giordano L., Sillo F., Garbelotto M., Gonthier P. (2018). Mitonuclear Interactions May Contribute to Fitness of Fungal Hybrids. Sci. Rep..

[69] Healy T.M., Burton R.S. (2023). Differential Gene Expression and Mitonuclear Incompatibilities in Fast- and Slow-Developing Interpopulation *Tigriopus californicus* Hybrids. Mol. Ecol..

[70] Liu W., Chen L., Zhang S., Hu F., Wang Z., Lyu J., Wang B., Xiang H., Zhao R., Tian Z. (2019). Decrease of Gene Expression Diversity during Domestication of Animals and Plants. BMC Evol. Biol..

[71] Liu F., Ma X.-B., Han B., Wang B., Xu J.-P., Cao B., Ling Z.-L., He M.-Q., Zhu X.-Y., Zhao R.-L. (2025). Pan-Genome Analysis Reveals Genomic Variations during Enoki Mushroom Domestication, with Emphasis on Genetic Signatures of Cap Color and Stipe Length. J. Adv. Res..

[72] Johnson M.S., Gopalakrishnan S., Goyal J., Dillingham M.E., Bakerlee C.W., Humphrey P.T., Jagdish T., Jerison E.R., Kosheleva K., Lawrence K.R. (2021). Phenotypic and Molecular Evolution across 10,000 Generations in Laboratory Budding Yeast Populations. eLife.

[73] Gallone B., Steensels J., Prahl T., Soriaga L., Saels V., Herrera-Malaver B., Merlevede A., Roncoroni M., Voordeckers K., Miraglia L. (2016). Domestication and Divergence of *Saccharomyces cerevisiae* Beer Yeasts. Cell.

