# Supplementary material for: Comparative Mitogenomics Reveals Intron Dynamics and Mitochondrial Gene Expression Shifts in Domesticated and Wild Pleurotus ostreatus
Source: J Fungi (Basel). 2026 Jan 20;12(1):75. doi: 10.3390/jof12010075 (PMC12842788; doi:10.3390/jof12010075)
Supplement: Supplementary file 1 [file jof-12-00075-s001.zip › jof-4089078-supplementary.pdf]

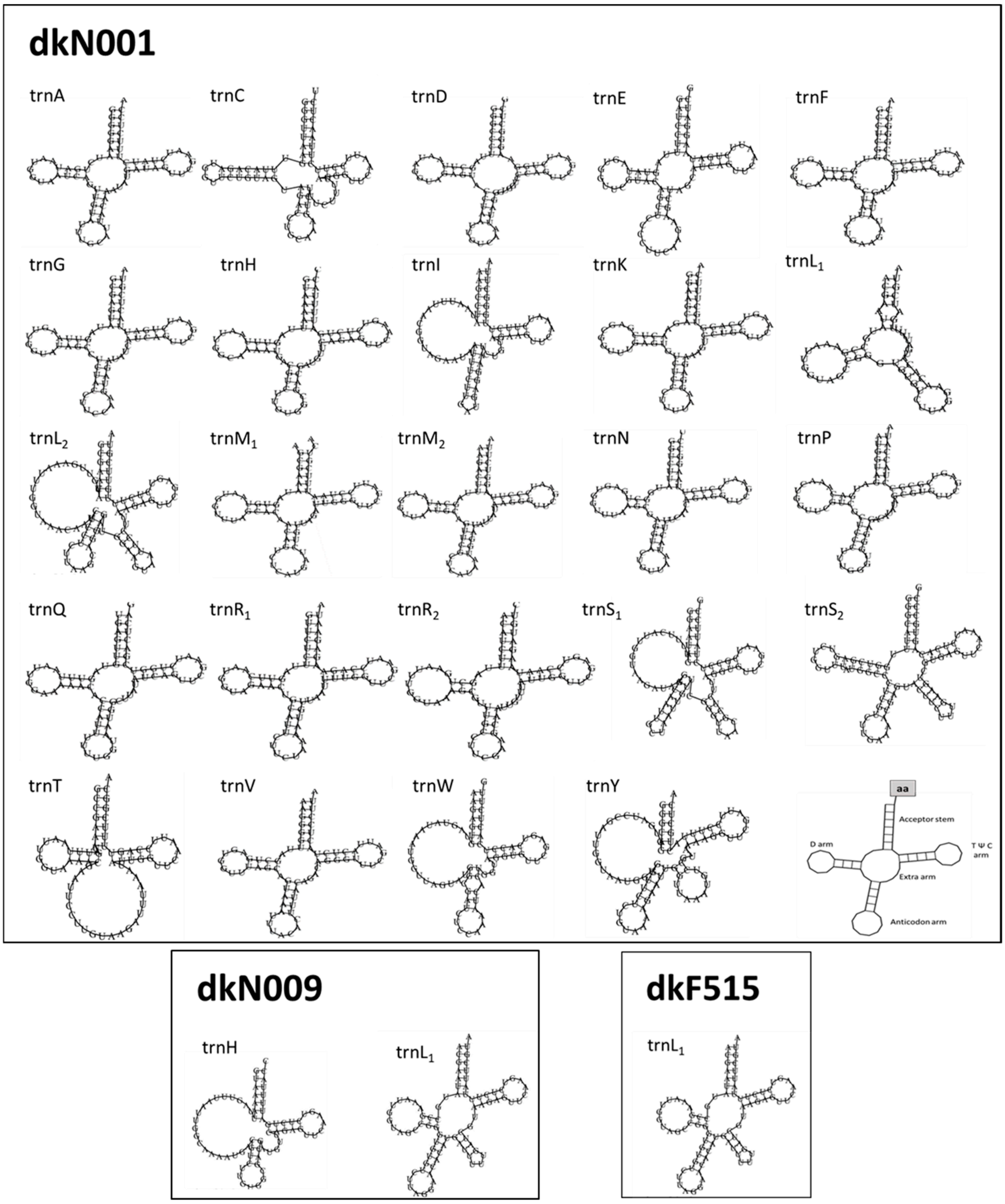

Figure S1: Predicted secondary structures of all tRNAs identified in the mitogenome of the dkN001 strain are shown. For the dkN009 and dkF515 strains, only the tRNA secondary structures that differ from those in dkN001 are presented.

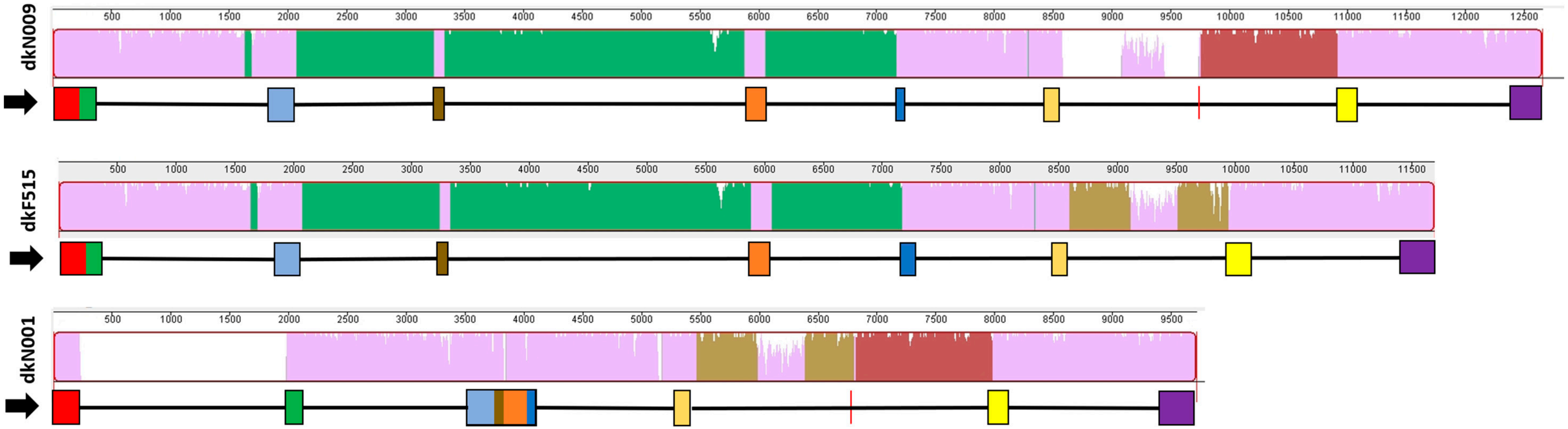

Figure S2: Structural alignments of the *cox1* sequences from dkN001, dkF515, and dkN009 based on analyses with the Mauve and MFannot tools. DNA homologous regions among strains identified with the Mauve tool are shown in the same colour and appear just below the bar indicating the gene length in base pairs. Arrows indicate exon and intron regions in the *cox1* sequences and are drawn to scale. Exons are represented as coloured blocks and the same colour denoting sequence homology. For example, exon 1 in dkF515 and dkN009 contains two regions (red and green). These two regions are separated in two exons in dkN001 by the presence of an intron. Black lines connecting the blocks represent intronic regions of the *cox1* gene.

a)

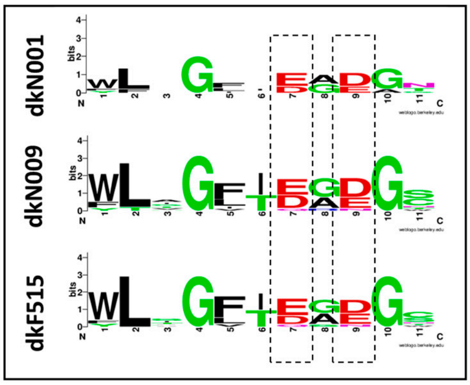

b)

Strains	Intron number	Intronic ORF	Conserved domain of HEGs	Number of repeat motives	motif I sequence	motif II sequence
dkN001	I1	*orf395*	LAGLIDADG_1	2	YLAGLIEGDGT	WLSGFSDADAN
	I2	*orf385*	LAGLIDADG_1	1	WLIGFTEAEGS	
	I4	*orf293*	LAGLIDADG_1	2	FFVGLLEGDGT	WLSGFIEAEGN
	I5	*orf378*	LAGLIDADG_1	1	WLAGIIDGDGN	
dkN009	I1	orf387	LAGLIDADG_1	2	WLVGFTDGEGT	WLIGFTEAEGS
	I2	orf329	LAGLIDADG_1	2	YLTGLIEGNGS	WLTGFIEADGC
	I3	orf317	LAGLIDADG_1	2	WLIGFTEGDGS	WLCGFTDREGC
	I3	orf305	LAGLIDADG_1	1	WLSGVIDGDGC	
	I4	orf336	LAGLIDADG_1	2	FLAGLIEGDGW	WLSGFTQADGC
	I6	orf278	LAGLIDADG_1	2	FTVGLIDGDGS	WLAGFIEAEGS
	I7	orf378	LAGLIDADG_1	1	WLAGIIDGDGN	
dkF515	I1	orf387	LAGLIDADG_1	2	WLVGFTDGEGT	WLIGFTEAEGS
	I2	orf329	LAGLIDADG_1	2	YLTGLIEGNGS	WLTGFIEADGC
	I3	orf317	LAGLIDADG_1	2	WLIGFTEGDGS	WLCGFTDSEGC
	I3	orf305	LAGLIDADG_1	1	WLSGVIDGDGC	
	I4	orf336	LAGLIDADG_1	2	FLAGLIEGDGW	WLSGFTQADGC
	I6	orf293	LAGLIDADG_1	1	WLSGFIEAEGN	

Figure S3: (a) Sequence logo of the putative LAGLIDADG_1 motifs located within introns of *cox1* gene *P. ostreatus* strains dkN001, dkN009 and dkF515. Conserved residues (D or E) are highlighted with dashed boxes. Information content at each position (in bits; X axis) is represented by the height of the amino acid. A score of 3.2 bits indicates high conservation, whereas a score of 0 implies low conservation. **(b) Alignment of the putative LAGLIDAD_1 motifs**. Amino acids are colour-coded according to chemical properties (blue, basic; red, acid; green, polar neutral; black hydrophobic).

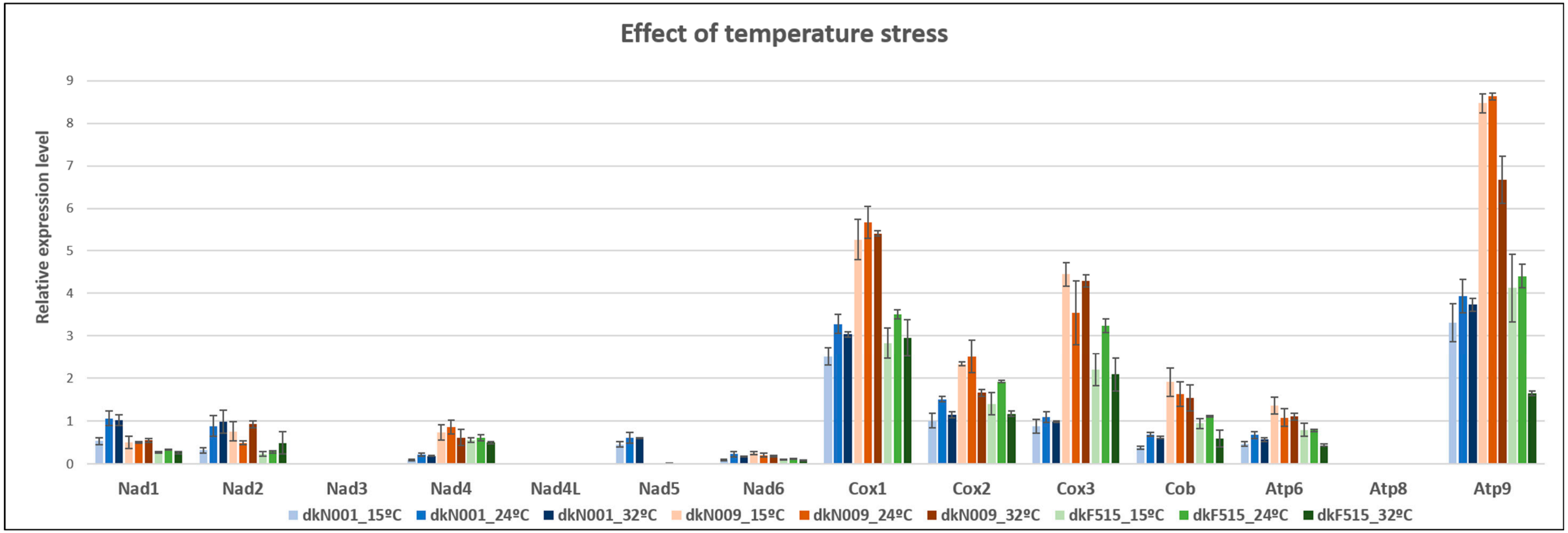

Figure S4: Relative expression of mitochondrial PCGs under SC (24ºC), and low-temperature (15ºC) and high-temperature (32ºC) stresses. Different lower letters indicate, in each PCG analysis, significant differences at level of P-value <0.05 according to Scheffe's test.

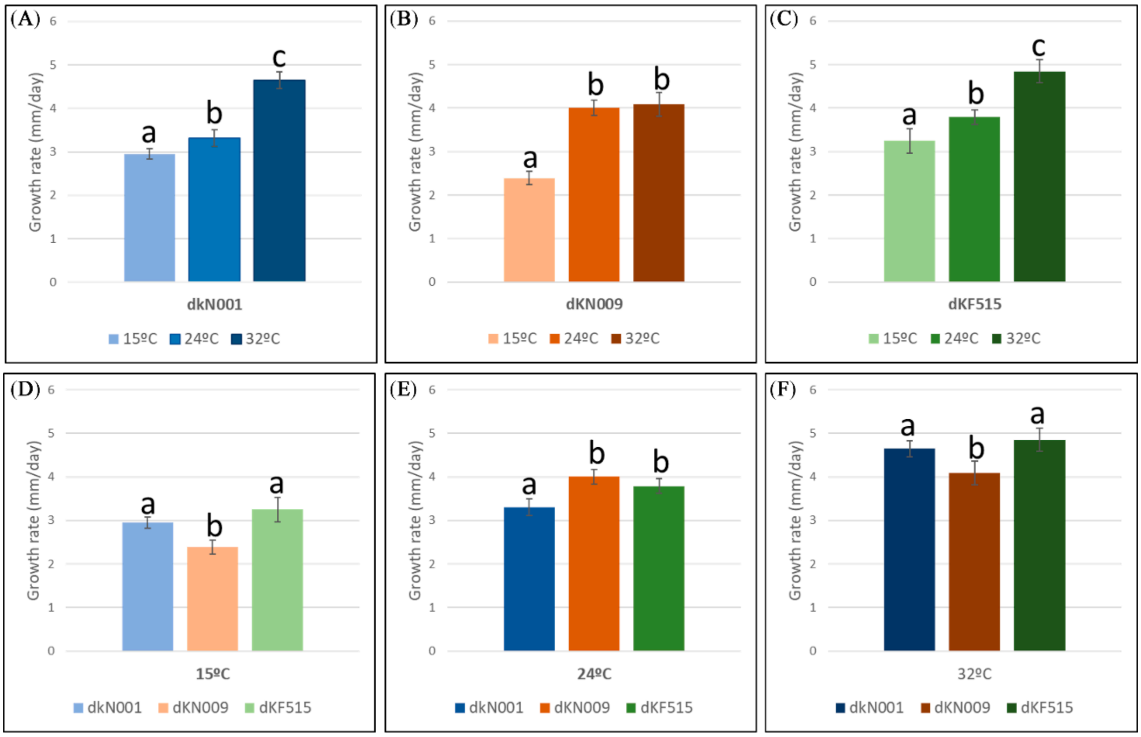

Figure S5: Growth rate of dikaryotic strains under SC (24ºC) conditions and at different temperatures. The figures (A), (B) and (C) represent the growth rate under 15ºC, 24º, and 32ºC of dkN001, dkN009 and dkF515, respectively. In contrast, the figures (D), (E) and (F) show the comparison of growth rate among strains at 15ºC, 24 ºC and 32ºC respectively. Different lower letters indicate, in each analysis, significant differences at level of P-value <0.05 according to Scheffe's test

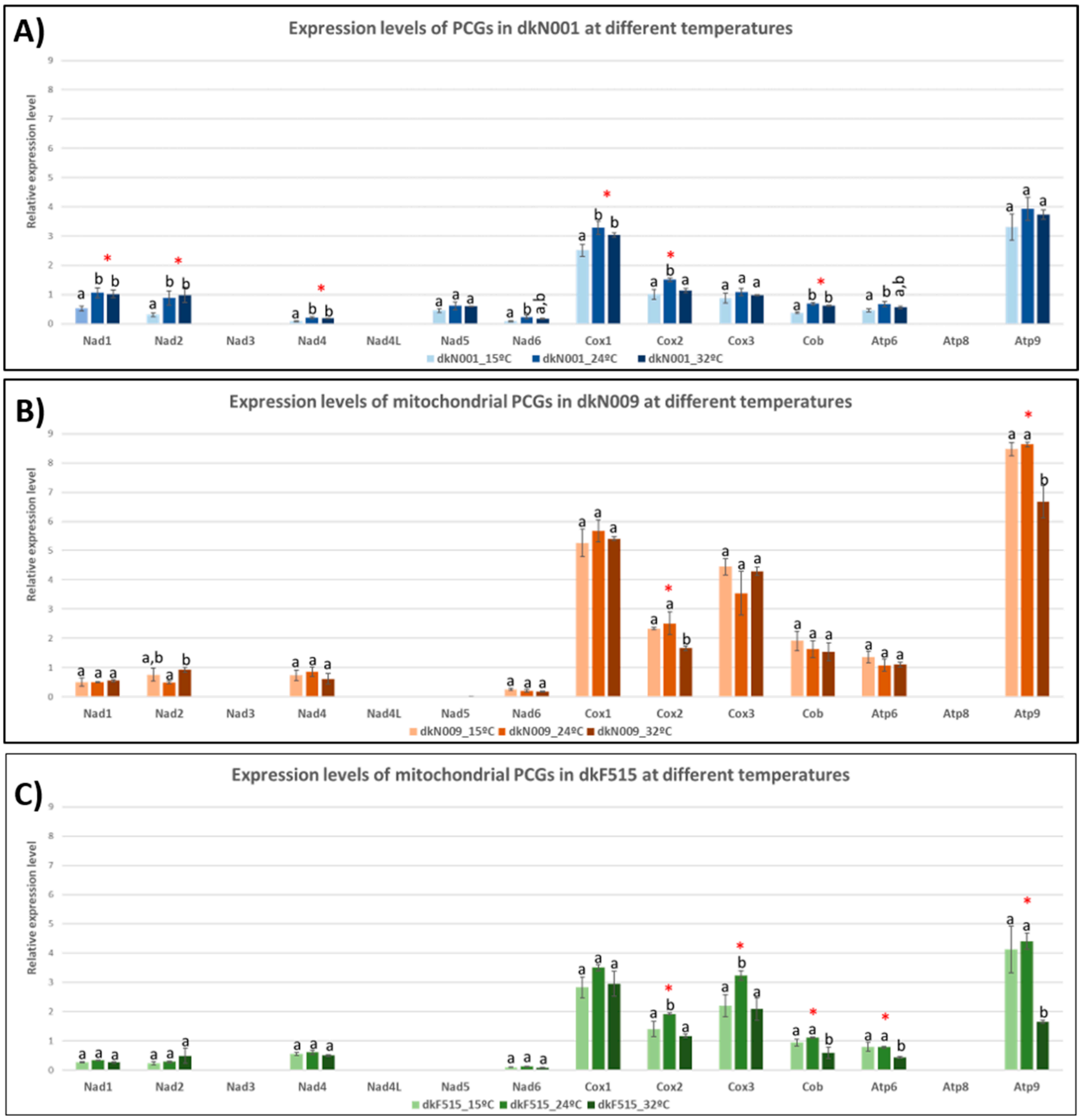

Figure S6: Expression levels of mitochondrial PCGs in (A) dkN001, (B) dkN009, and (C) dkF515 strains at 15, 24 and 32ºC. Red asterisks indicate mitochondrial PCGs showing significant differences in expression levels across temperatures. Different lower letters indicate, in each analysis, significant differences at level of P-value <0.05 according to Scheffe's test.

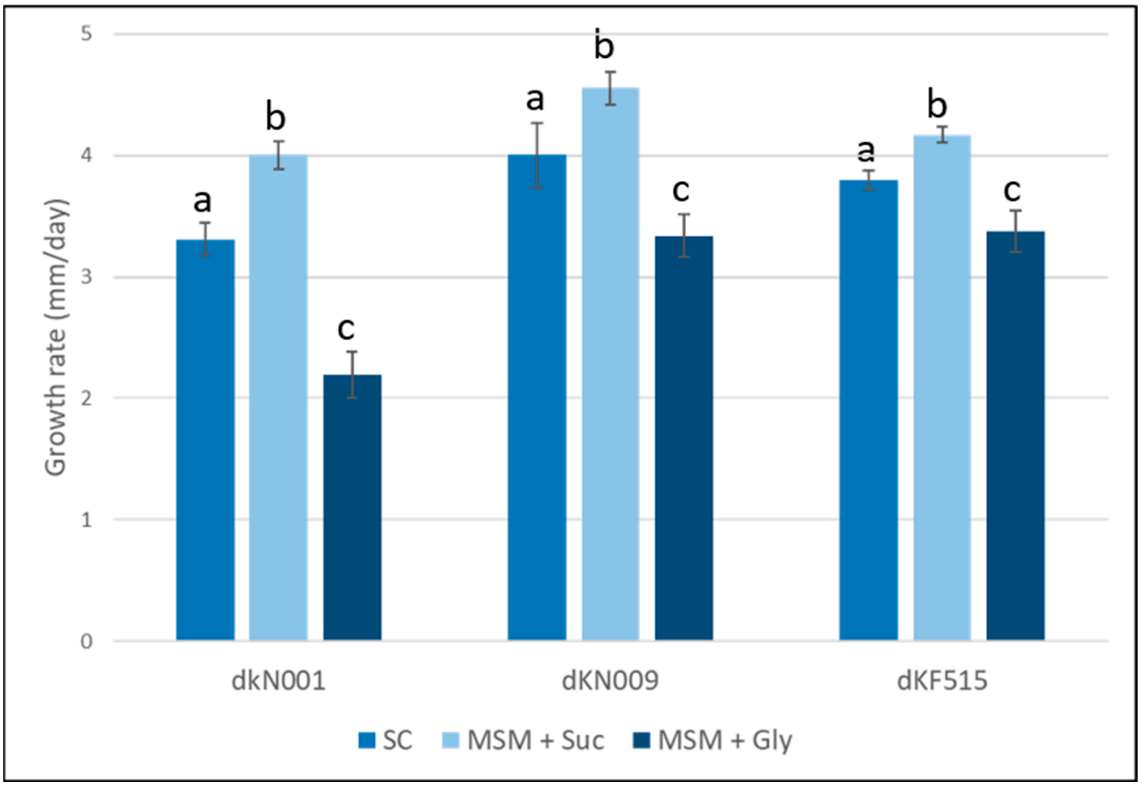

Figure S7: Growth rate of dikaryotic strains under SC (MESM medium) conditions and with different carbon sources. (A) Growth rate of dkN001, dkN009, and dkF515 under SC (MESM) and MSM supplemented with sucrose, and glycerol. Different lowercase letters indicate significant differences at level of P-value <0.05 according to Scheffe's test.

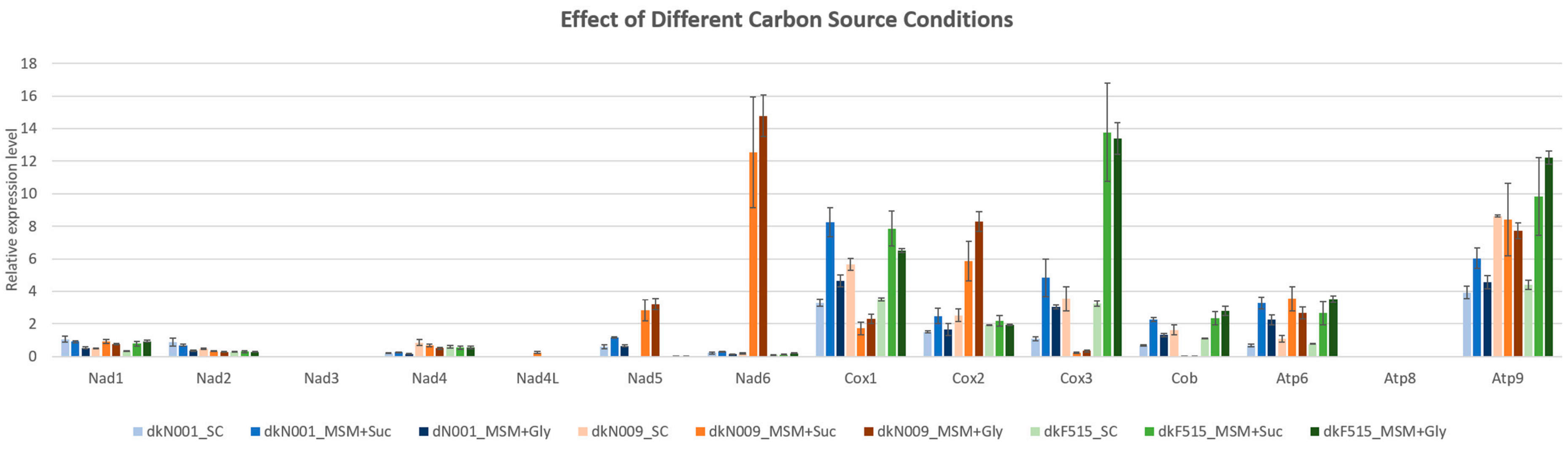

Figure S8: Relative expression of mitochondrial PCGs under different carbon sources conditions: SC (MESM), MSM supplemented with 1% sucrose and MSM supplemented with 1% glycerol. Different lower letters indicate, in each PCG analysis, significant differences at level of P-value <0.05 according to Scheffe's test.

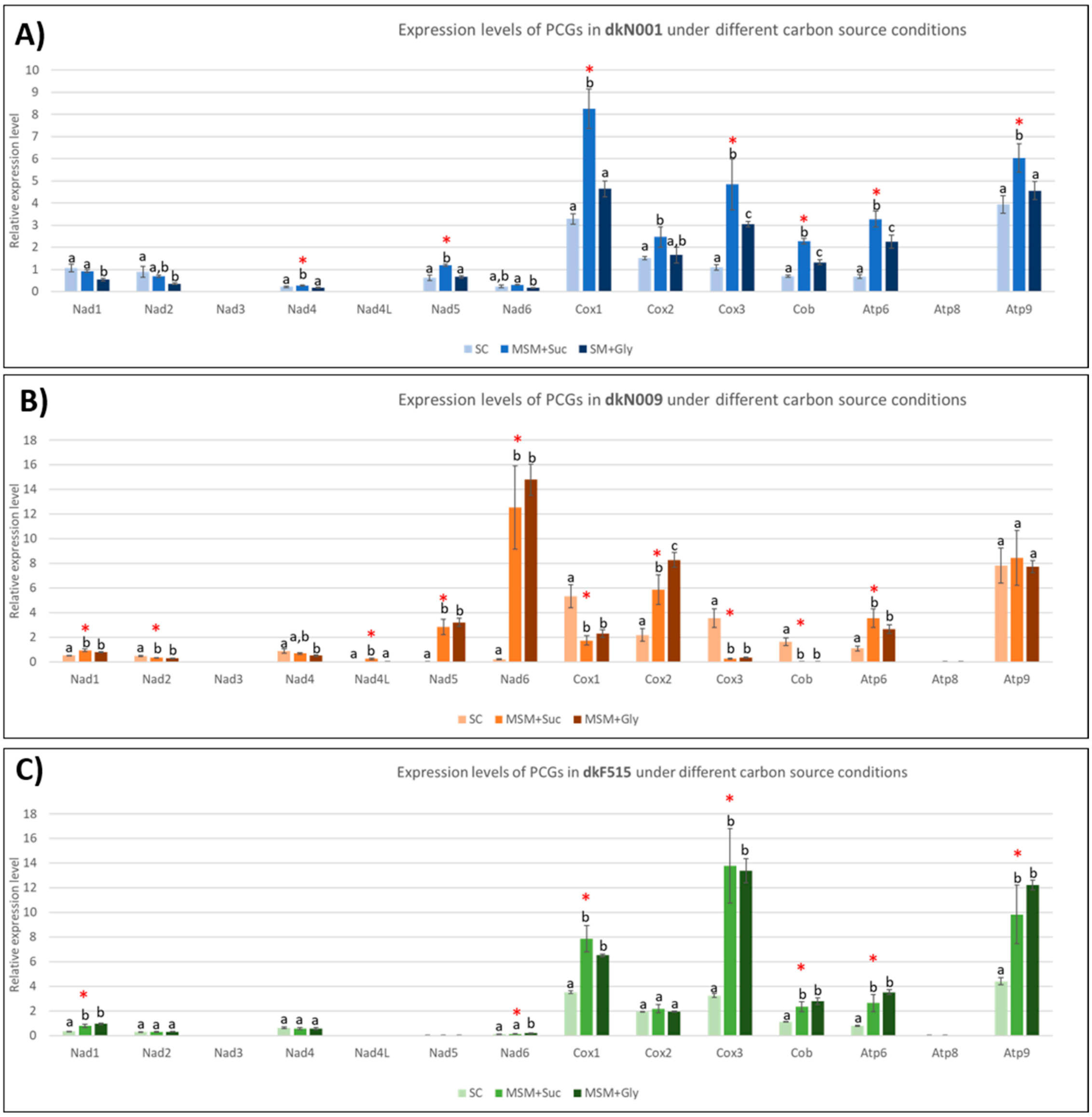

Figure S9: Expression levels of mitochondrial PCGs in the dikaryotic strains (A) dkN001, (B) dkN009 and (C) dkF515 grown under different carbon sources. Red asterisks indicate mitochondrial PCGs showing significant differences in expression levels across different carbon source conditions.

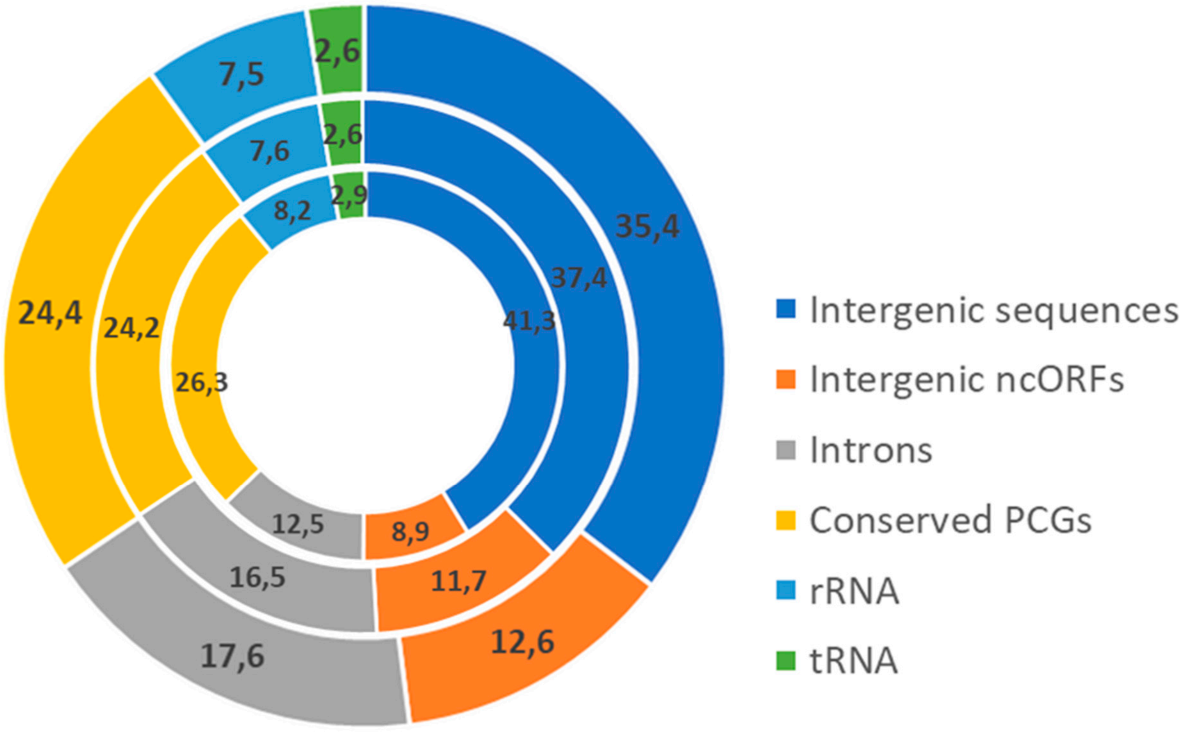

Figure S10: Proportion (%) of different components in the mitogenomes of dkN001, dkF515, and dkN009 strains of *P. ostreatus*. The inner circle corresponds to dkN001, the middle circle to dkF515, and the outer circle to dkN009. The intergenic ncORFs correspond to hypothetical protein-coding sequences identified by MFannot program.
